# Built environmental characteristics and diabetes: a systematic review and meta-analysis

**DOI:** 10.1186/s12916-017-0997-z

**Published:** 2018-01-31

**Authors:** N. R. den Braver, J. Lakerveld, F. Rutters, L. J. Schoonmade, J. Brug, J. W. J. Beulens

**Affiliations:** 1 Department of Epidemiology & Biostatistics, Amsterdam Public Health Research Institute, VU University Medical Center, De Boelelaan 1089a, 1081HV Amsterdam, The Netherlands; 2 University Library, VU, Amsterdam, The Netherlands; 3 Amsterdam School for Communication Research, University of Amsterdam, Amsterdam, The Netherlands; 4 Julius Center for Health Sciences and Primary Care, University Medical Center Utrecht, Utrecht, The Netherlands

**Keywords:** Built environment, Type 2 diabetes mellitus, Lifestyle behaviour, Prevention, Urbanisation

## Abstract

**Background:**

The built environment influences behaviour, like physical activity, diet and sleep, which affects the risk of type 2 diabetes mellitus (T2DM). This study systematically reviewed and meta-analysed evidence on the association between built environmental characteristics related to lifestyle behaviour and T2DM risk/prevalence, worldwide.

**Methods:**

We systematically searched PubMed, EMBASE.com and Web of Science from their inception to 6 June 2017. Studies were included with adult populations (>18 years), T2DM or glycaemic markers as outcomes, and physical activity and/or food environment and/or residential noise as independent variables. We excluded studies of specific subsamples of the population, that focused on built environmental characteristics that directly affect the cardiovascular system, that performed prediction analyses and that do not report original research. Data appraisal and extraction were based on published reports (PROSPERO-ID: CRD42016035663).

**Results:**

From 11,279 studies, 109 were eligible and 40 were meta-analysed. Living in an urban residence was associated with higher T2DM risk/prevalence (*n* = 19, odds ratio (OR) = 1.40; 95% CI, 1.2–1.6; *I*^2^ = 83%) compared to living in a rural residence. Higher neighbourhood walkability was associated with lower T2DM risk/prevalence (*n* = 8, OR = 0.79; 95% CI, 0.7–0.9; *I*^2^ = 92%) and more green space tended to be associated with lower T2DM risk/prevalence (*n* = 6, OR = 0.90; 95% CI, 0.8–1.0; *I*^2^ = 95%). No convincing evidence was found of an association between food environment with T2DM risk/prevalence.

**Conclusions:**

An important strength of the study was the comprehensive overview of the literature, but our study was limited by the conclusion of mainly cross-sectional studies. In addition to other positive consequences of walkability and access to green space, these environmental characteristics may also contribute to T2DM prevention. These results may be relevant for infrastructure planning.

**Electronic supplementary material:**

The online version of this article (doi:10.1186/s12916-017-0997-z) contains supplementary material, which is available to authorized users.

## Background

Key risk factors for type 2 diabetes mellitus (T2DM) are lack of physical activity, an unhealthy diet and lack of sleep [1, 2]. Real-life T2DM prevention programmes aimed at changing people’s lifestyle and behaviour have often been ineffective in the long term [3]. An important reason for this may be the focus on individual-level determinants of these lifestyle behaviours, such as motivation and ability, whereas they are also determined by more upstream drivers, such as the availability and accessibility of healthy options in an individual’s environment. In terms of changing and sustaining healthy lifestyle behaviours, the built environment is of importance [4–7].

Urbanisation is one example of an upstream driver. Urbanisation is associated with lower total physical activity and increased consumption of processed foods, which are high in fat, added sugars, animal products and refined carbohydrates [4, 8]. However, urbanisation has also been linked to higher total walking and cycling for transportation [4]. Built environmental characteristics, such as higher walkability, access to parks, and access to shops and services, are consistently associated with higher physical activity [4, 5]. Food built environmental characteristics, such as the perceived availability of healthy foods, are also associated with higher diet quality. In addition, greater availability of fast-food outlets has been associated with lower fruit and vegetable consumption [9, 10]. Other built environmental characteristics have been associated with higher stress and lack of sleep through residential noise, e.g. noise due to road and air traffic [11, 12].

By influencing physical activity, diet and sleep, these built environmental characteristics may also affect the risk/prevalence of T2DM. Indeed, the diabetes atlas showed higher T2DM prevalence in urban vs. rural areas [8], and a recent systematic meta-analysis reported similar results for South East Asia [13]. Two other systematic reviews addressed the association between specific built environmental characteristics and T2DM [14, 15]. However, one review only included German studies [14], while the second review included a broad range of cardiovascular disease outcomes, but only one study was included that considered T2DM as an outcome [15]. A recent meta-analysis showed that higher residential noise was associated with higher T2DM risk [16].

A comprehensive systematic review and meta-analysis of the current international evidence is, thus, lacking. This study aims to review systematically the evidence on the association between built environmental characteristics related to lifestyle behaviours and T2DM risk or prevalence, worldwide. Since characteristics of the built environment may vary with the country-specific income level, we stratified our analyses by this factor when possible. Meta-analyses were performed when three or more studies investigated the same exposure and outcome.

## Methods

### Data sources and searches

A literature search was performed based on the Preferred Reporting Items for Systematic Reviews and Meta-Analysis (PRISMA) statement (www.prisma-statement.org). We systematically searched the bibliographic databases PubMed, EMBASE.com and Web of Science Core Collection from their inception to 6 June 2017 (NdB and LS). Search terms included indexed terms from MeSH in PubMed, EMtree in EMBASE.com, as well as free-text terms. We used free-text terms only in Web of Science. Search terms expressing ‘diabetes’ were used in combination with search terms comprising ‘environment’. Bibliographies of the identified articles were hand-searched for relevant publications. Duplicate articles were excluded. The full search strategies for all databases can be found in Additional file 1. The protocol and search strategy used were uploaded to PROSPERO prior to the study being carried out (CRD42016035663).

### Study selection

Two reviewers independently screened titles, abstracts and full-text articles for eligibility (NdB and JL, or JWJB). Studies were included if they: (i) studied a population of adults, 18 years or older; (ii) had T2DM incidence or prevalence, or the glycaemic markers HbA1c, glucose or insulin sensitivity as outcomes; (iii) included independent variables covering built environmental characteristics that potentially influence the risk of T2DM via lifestyle behaviours, physical activity, diet and sleep; and (iv) were written in English, Dutch or German. We excluded studies if they: (i) were not conducted in the general population, but in specific subsamples, like pregnant women, or T2DM patients; (ii) focused on built environmental characteristics that directly affect the cardiovascular system (i.e. not via lifestyle behaviours), such as exposure to particulates due to roadway proximity; (iii) performed prediction analyses or (iv) were specific publication types that do not report original scientific research (editorials, letters, legal cases and interviews). As in the general population, the vast majority of diabetes cases are T2DM (>90%), studies were included if they did not specify the type of diabetes (type 1 diabetes mellitus or T2DM). Inconsistencies in study selection were resolved through consensus with a third reviewer (JL or JWJB).

### Data extraction

One reviewer (NdB) performed data extraction, according to a standard protocol, including measures of study design, outcome, outcome assessment and exposure assessment, demographics, and prevalence or effect measure. Data extraction was appraised by a second reviewer (JL) for a random subsample of the included studies.

### Quality assessment

Two reviewers (NdB and JWJB, or JL) independently evaluated the methodological quality of the full-text papers using the Quality Assessment Tool for Quantitative Studies, as described earlier by Mackenbach et al. [17]. This tool provides a quality score based on study design, representativeness at baseline (selection bias) and follow-up (withdrawals and drop-outs), confounders, data collection, data analysis and reporting. Each domain received a weak, moderate or strong score, resulting in seven scores. A study was rated as strong when it received four strong ratings and no weak ratings. A study was rated as moderate if it received one weak rating and less than four strong ratings. Finally, a study was rated weak if it received two or more weak ratings. Study quality was assessed in terms of the reported association between the relevant built environmental characteristic and T2DM, even if this was not the primary analysis presented in the study. Studies with a weak rating (*n* = 23) are presented in Additional file 2 and were included in sensitivity analyses, but excluded from the main analyses.

### Data synthesis

Study characteristics were described in a systematic manner, according to the built environmental characteristics under investigation. These categories were made as homogeneous as possible, based on the lifestyle behaviours. Findings were further described according to country-level income, based on the World Bank list of economies, 2016 [18].

Studies were meta-analysed when three or more studies investigated the same exposure and outcome variables. In addition, the studies had to provide at least age and sex adjusted or standardised risk ratios or prevalence, and have a moderate or strong quality rating. If reported ratios were stratified and could not be pooled with the information provided in the publication, the study’s authors were contacted and asked to provide the pooled-risk ratio [19–23]. Reference categories were harmonised by taking the inverse of the risk ratio and 95% confidence interval (CI). If a risk ratio for a continuous variable was reported, we transformed this to a categorical risk ratio based on the methods of Danesh et al. [24]. Forest plots and random-effects meta-analysis models were fitted to relative risks or odds ratios. Plots and models were stratified for country income level and study quality, where permitted. In the sensitivity analyses, the studies with weak quality ratings were added to the models. Heterogeneity was tested using *I*^2^. Analyses were performed in R version 3.2.5 using the Metafor package.

## Results

**Fig. 1:**
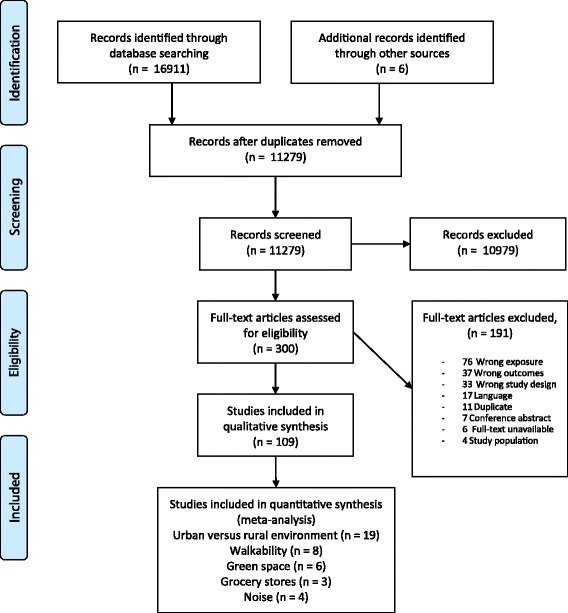
Flow chart of study inclusion

From the 11,279 identified references, 299 full articles were screened, and 109 of these studies were included in our review, of which 23 were not included in our main analyses due to a weak quality rating (Fig. 1 and Additional file 2). Included studies were categorised according to the built environmental characteristic investigated (Tables 1 and 2), and built environments were subdivided by physical activity environment, food environment and residential noise (Table 2).

**Table 1:** Study characteristics and results of studies investigating the association between urban and rural built environments and diabetes mellitus

Author	Year	Country	Country income level	Study design	Sample size	Age	Outcome^a^	Outcome assessment^b^	Result			Adjustment for confounding	Quality statement
									Urban > rural	Rural > urban	No difference		
Aekplakorn et al. [89]	2011	Thailand	Upper middle	Cross-sectional	18,629	NFG: 44.3 ± 0.3 Diabetes mellitus: 54.1 ± 0.7	T2DM/T1DM prevalence	Blood sample	X			Age, sex	Moderate
Agyemang et al. [90]	2016	Ghana, Netherlands, Germany, England	Lower middle and high	Cross-sectional	5659	25–70 years (NR)	T2DM prevalence	Blood sample	X			Age, sex, education	Moderate
Ali et al. [91]	1993	Malaysia	Upper middle	Cross-sectional	681	38.6 ± 13.7	T2DM/T1DM prevalence	Blood sample			X	Age	Moderate
Al-Moosa et al. [92]	2006	Oman	High	Cross-sectional	5840	24% >50 years 41% < 30 years	T2DM/T1DM prevalence	Blood sample	X			–	Moderate
Anjana et al. [93]	2011	India	Lower middle	Cross-sectional	13,055	40 ± 14	T2DM/T1DM prevalence	Blood sample	Southern area, western area, eastern area		Northern area	Age, sex	Moderate
Assah et al. [94]	2011	Cameroon	Lower middle	Cross-sectional	552	38.4 ± 8.6	T2DM/T1DM prevalence	Blood sample	X			–	Moderate
Attard et al. [67]	2012	China	Upper middle	Cross-sectional	NA	51 ± 0.4	T2DM/T1DM prevalence	Blood sample, self-report	X			Age, sex, income, region, BMI	Strong
Allender et al. [95]	2011	Sri Lanka	Lower middle	Cross-sectional	4485	46.1 ± 15.1	T2DM/T1DM prevalence	Blood sample	X			Age, sex, income	Moderate
Bahendeka et al. [41]	2016	Uganda	Low	Cross-sectional	3689	35.1 ± 12.6	T2DM/T1DM prevalence	Blood sample			X	Age, sex, region of residence, floor finishing of dwelling, BMI, waist circumference, total cholesterol	Moderate
Baldé et al. [96]	2007	Guinea	Low	Cross-sectional	1537	47.7 ± 12.5	T2DM/T1DM prevalence	Blood sample	X			Age, location, excess of waist, raised systolic BP, raised diastolic BP	Moderate
Balogun et al. [97]	2012	Nigeria	Lower middle	Longitudinal	1330	77.3 ± 0.3	T2DM incidence	Self-report	X			Age, sex, education	Strong
Baltazar et al. [98]	2003	Philippines	Lower middle	Cross-sectional	7044	39.0 ± 0.5	T2DM/T1DM prevalence	Blood sample			X	Age and sex	Moderate
Barnabé-Ortiz [99]	2016	Peru	Upper middle	Longitudinal	3123	24% < 45 years 25% >65 years	T2DM incidence	Blood sample			X	Sex, age, education level, SES, family history of diabetes, daily smoking, hazardous drinking, TV watching for 2+ hours per day, transport-related physical inactivity, fruit and vegetable consumption, BMI, metabolic syndrome	Moderate
Bocquier et al. [100]	2010	France	High	Cross-sectional	3,038,670	48.9 ± 18.6	T2DM/T1DM prevalence	Secondary	X			Age, sex	Strong
Cubbin et al. [23]	2006	Sweden	High	Cross-sectional	18,081	48% >45 years 25% < 35 years	T2DM/T1DM prevalence	Self-report		X		Age, sex, marital status, immigration status, SES composite, neighbourhood deprivation	Moderate
Christensen et al. [101]	2009	Kenya	Lower middle	Cross-sectional	1459	38.6 ± 12.6	T2DM/T1DM prevalence	Blood sample	X			Age, sex	Moderate
Dagenais et al. [102]	2016	Bangladesh, India, Pakistan, Zimbabwe, China, Colombia, Iran, Argentina, Brazil, Chile, Malaysia, Poland, South Africa, Turkey, Canada, Sweden, United Arab Emirates	Lower, lower middle, upper middle and high	Cross-sectional	119,666	52 ± 9.3	T2DM/T1DM prevalence	Blood sample	X			Age, sex, residency location, BMI, waist-to-hip ratio, PA levels, AHEI score, combined former and current smoking, education level, family history of diabetes, ethnicity	Strong
Dar et al. [25]	2015	India	Lower middle	Cross-sectional	3972	43% >50 years 57% 40–50 years	T2DM prevalence	Blood sample	X			–	Weak
Davila et al. [103]	2013	Colombia	Upper middle	Cross-sectional	1026	35% >55 years 35% < 35 years	T2DM/T1DM prevalence	Blood sample			X	Age, sex, education, SES, marital status, smoking, alcohol, intake of fruit and vegetables, PA	Strong
Delisle et al. [104]	2012	Benin	Low	Cross-sectional	541	38.2 ± 0.6	Glycaemic marker: HOMA index	Blood sample	X			Age, sex, SES, location, diet quality, PA, alcohol, BMI	Moderate
Dong et al. [105]	2005	China	Upper middle	Cross-sectional	12,240	46.4 ± 13.9	T2DM prevalence	Blood sample	X (men)		X (women)	Age, sex	Moderate
Du et al. [106]	2016	China	Upper middle	Cross-sectional	3797	15% >60 years 8% 20–29 years	T2DM/T1DM prevalence	Blood sample			X	Age, sex	Moderate
Esteghamati et al. [107]	2009	Iran	Upper middle	Cross-sectional	3397	23% >55 years 25% < 35 years	T2DM/T1DM prevalence	Blood sample			X	Age, sex, residential area	Moderate
Georgousopoulou et al. [108]	2017	Mediterranean islands	High	Cross-sectional	2749	75 ± 7.3	T2DM/T1DM prevalence	Blood sample	X			Age, sex, BMI, physical inactivity, smoking, siesta habit, education, living alone, adherence to Mediterranean diet, GDS, number of friends and family members, frequency of going out with friends and family, number of holiday excursions per year	Moderate
Gong et al. [109]	2015	China	Upper middle	Cross-sectional	5923	38% >50 years 62% < 50 years	T2DM/T1DM prevalence	Blood sample	X			Age, sex, education, PA, smoking, alcohol, BMI, triglycerides, HDL-cholesterol, hypertension	Strong
Hussain et al. [110]	2004	Bangladesh	Lower middle	Cross-sectional	6312	14% >50 years 46% < 30 years	T2DM/T1DM prevalence	Blood sample	X			Age, sex	Moderate
Han et al. [111]	2017	Korea	High	Longitudinal	7542	52 ± 8.8	T2DM incidence	Blood sample	X			Age, sex, residential area, family history of diabetes, smoking, alcohol, exercise, abdominal obesity, hypertension, high triglycerides, low HDL-cholesterol	Strong
Katchunga et al. [112]	2012	Congo	Low	Cross-sectional	699	42.5 ± 18.1	T2DM/T1DM prevalence	Blood sample			X	–	Moderate
Keel et al. [113]	2017	Australia	High	Cross-sectional	4836	Non-indigenous: 66.6 ± 9.7 Indigenous: 54.9 ± 8.7	T2DM/T1DM prevalence	Self-report		X (indigenous)	X (non-indigenous)	Age, sex, ethnicity, education, English-speaking at home, ethnicity	Moderate
Mayega et al. [114]	2013	Uganda	Low	Cross-sectional	1497	45.8% >45 years 54.2% < 45 years	T2DM prevalence	Blood sample		X		Age, sex, residence, occupation, family history of diabetes, BMI, PA level, dietary diversity	Strong
Mohan et al. [115]	2016	India	Lower middle	Cross-sectional	6853	35–70 years (NR)	T2DM/T1DM prevalence	Blood sample	X			Age (only women included)	Moderate
Msyamboza et al. [116]	2014	Malawi	Low	Cross-sectional	3056	12.5% >55 years 45% < 35 years	T2DM/T1DM prevalence	Blood sample			X	Age, sex	Moderate
Ntandou et al. [117]	2009	Benin	Low	Cross-sectional	541	38.2 ± 10	T2DM/T1DM prevalence	Blood sample		X		Age, sex, waist circumference, education, SES, PA, micronutrient adequacy score, preventive diet score, alcohol	Moderate
Oyebode et al. [118]	2015	China, Ghana, India, Mexico, Russia, South Africa	Upper and Lower middle	Cross-sectional	39,436	47.3% >60 years 12.3% < 40Y	T2DM/T1DM prevalence	Self-report	X (pooled)			Age, sex, survey design, income quintile, marital status, education	Strong
Papoz et al. [119]	1996	New Caledonia	High	Cross-sectional	9390	30–59 years (NR)	T2DM/T1DM prevalence	Blood sample	X			Age	Moderate
Pham et al. [120]	2016	Vietnam	Lower middle	Cross-sectional	16,730	54 ± 8	T2DM/T1DM prevalence	Blood sample	X (men)		X (women)	Age, sex, socio-demographic factors, anthropometric measures, BP, family history of diabetes	Moderate
Raghupathy et al. [121]	2007	India	Lower middle	Longitudinal	2218	28 ± 1.2	T2DM prevalence	Blood sample	X			Age, sex, number of household possessions, education, PA, smoking, alcohol, parental consanguinity, family history of diabetes mellitus, body fat, BMI, waist-to-hips ratio, subscapular/triceps ratio	Strong
Ramdani et al. [122]	2012	Morocco	Lower middle	Cross-sectional	1628	54.2 ± 10.9	T2DM/T1DM prevalence	Blood sample			X	Age, sex, BMI	Moderate
Sadikot et al. [123]	2004	India	Lower middle	Cross-sectional	41,270	36% >50 years 34% < 40 years	T2DM prevalence	Blood sample	X			Age, sex	Moderate
Sobngwi et al. [124]	2004	Cameroon	Lower middle	Longitudinal	1726	24% >55 years 28% < 35 years	T2DM/T1DM prevalence	Blood sample	X (women)		X (men)	Age, sex, residence, socio-professional category, alcohol, smoking, PA	Moderate
Stanifer et al. [125]	2016	Tanzania	Low	Cross-sectional	481 neighbourhoods	25% >60 years	T2DM/T1DM prevalence	Blood sample			X	Age, sex	Moderate
Weng et al. [126]	2007	China	Upper middle	Cross-sectional	529	NR	T2DM/T1DM prevalence	Blood sample			X	Age, sex	Moderate
Wu et al. [127]	2016	China	Upper middle	Cross-sectional	23,010	40 (30.4–56.3)	T2DM/T1DM prevalence	Blood sample			X	Age	Moderate
Zhou et al. [128]	2015	China	Upper middle	Cross-sectional	98,658	20% >60 years 80% < 60 years	T2DM/T1DM prevalence		X			Age, sex, region	Moderate

*BMI* body mass index, *BP* blood pressure, *NR* not recorded, *PA* physical activity, *SES* socioeconomic status, *T1DM* type 1 diabetes mellitus, *T2DM* type 2 diabetes mellitus, *NFG* normal fasting glucose, *HOMA* homeostasis model assessment, *GDS* geriatric depression scale

^a^Prevalence indicates incidence or glycaemic marker level

^b^Blood sample: study diagnosed diabetes based on glycaemic marker or oral glucose tolerance test; secondary: from data sources such as national health survey; self-report: ever diagnosed with diabetes

**Table 2:** Study characteristics of studies investigating physical activity environment, food environment, residential noise and diabetes mellitus

Author	Year	Country	Income level	Study design	Sample size	Age	Outcome^a^	Outcome assessment^b^	Exposure category	Exposure assessment	Level geodata	Quality statement
Ahern et al. [46]	2011	US	High	Cross-sectional	3128	NR	T2DM/T1DM prevalence	Secondary	PA, food	Place of residence	Aggregate	Moderate
AlHasan et al. [69]	2016	US	High	Cross-sectional	NA	NR	T2DM/T1DM prevalence	Secondary	Food	GIS	Aggregate	Strong
Astell-Burt et al. [42]	2014	Australia	High	Cross-sectional	48,072	28% 45–55 years 39% >65 years	T2DM/T1DM prevalence	Self-report	PA	GIS	Individual	Moderate
Auchincloss et al. [47]	2009	US	High	Longitudinal	2285	62.1 ± 10	T2DM incidence	Blood sample, self-report	PA, food	Self-report	Individual	Moderate
Bodicoat et al. [44]	2014	UK	High	Cross-sectional	10,476	59 ± 10.4	T2DM prevalence	Secondary (screen detected)	PA	GIS	Individual	Strong
Bodicoat et al. [72]	2015	UK	High	Cross-sectional	10,461	59 ± 10.4	T2DM prevalence	Secondary (screen detected)	Food	GIS	Individual	Strong
Booth et al. [19]	2013	Canada	High	Longitudinal	1,024,380	30–64 years (NR)	T2DM/T1DM incidence	Secondary	PA			Moderate
Braun et al. [80]	2015	US	High	Cross-sectional	NA	NR	T2DM/T1DM prevalence	Secondary	PA, food	Register	Aggregate	Moderate
Braun et al. [58]	2016	US	High	Longitudinal	1079	39.7 ± 3.7	Glycaemic marker: ln(HOMA index)	Blood sample	PA	GIS	Individual	Strong
Braun et al. [57]	2016	US	High	Longitudinal	583	69.4 ± 9.5	Glycaemic marker: fasting glucose	Blood sample	PA	GIS	Individual	Strong
Cai et al. [82]	2017	Netherlands	High	Cross-sectional	93,277	44.9 ± 12.3	Glycaemic marker: fasting glucose	Blood sample	Noise	GIS	Aggregate	Strong
Carroll et al. [71]	2017	Australia	High	Longitudinal	2582	50 ± 15	Glycaemic marker: HbA1c	Blood sample	Food	GIS	Aggregate	Moderate
Christine et al. [48]	2015	US	High	Longitudinal	2157	60.7 ± 9.9	T2DM incidence	Blood sample	PA, food	GIS, self-report	Individual	Strong
Creatore et al. [20]	2016	Canada	High	Longitudinal	±4,505,000	61% 30–49 years 34% 50–65 years	T2DM/T1DM incidence	Secondary	PA	GIS	Aggregate	Strong
Cunningham-Myrie et al. [49]	2015	Jamaica	Upper middle	Cross-sectional	2848	36.9 ± 2.7	T2DM/T1DM prevalence	Blood sample	PA	Environmental audit	Individual	Strong
Dalton et al. [59]	2016	UK	High	Longitudinal	23,865	59.1 ± 9.3	T2DM/T1DM incidence	Self-report	PA	GIS	Individual	Strong
Dzhambov et al. [83]	2016	Bulgaria	Upper middle	Cross-sectional	581	36.5 ± 15.4	T2DM/T1DM prevalence	Secondary	Noise	Secondary	Aggregate	Moderate
Eichinger et al. [50]	2015	Austria	High	Cross-sectional	660	47.1 ± 14.1	T2DM/T1DM prevalence	Blood sample	PA	Self-report	Individual	Moderate
Eriksson et al. [85]	2014	Sweden	High	Longitudinal	5156	47 ± 5	T2DM incidence	Blood sample	Noise	GIS	Individual	Moderate
Flynt et al. [73]	2015	US	High	Cross-sectional	NA	NR	T2DM/T1DM prevalence	Secondary	Food	Secondary	Aggregate	Moderate
Frankenfeld et al. [74]	2015	US	High	Cross-sectional	3227	11% >65 years 75% >18 years	T2DM/T1DM prevalence	Blood sample	Food	GIS	Aggregate	Moderate
Freedman et al. [68]	2011	US	High	Cross-sectional	NA	100% >50 years	T2DM/T1DM prevalence	Self-report	PA, food	Secondary	Aggregate	Moderate
Fujiware et al. [60]	2017	Japan	High	Cross-sectional	8904	72.5 ± 5.2	T2DM/T1DM prevalence	Blood sample	PA, food	GIS	Individual	Moderate
Gebreab et al. [61]	2017	US	High	Longitudinal	3661	54 ± 12	T2DM incidence	Blood sample	PA, Food	GIS	Individual	Strong
Glazier et al. [21]	2014	Canada	High	Cross-sectional	2,446,029		T2DM/T1DM prevalence	Secondary	PA	GIS	Aggregate	Moderate
Hipp et al. [78]	2015	US	High	Cross-sectional	3109 counties		T2D prevalence	Secondary	Food	GIS	Aggregate	Moderate
Heideman et al. [86]	2014	Germany	High	Longitudinal	3604	44.8 ± 13.7	T2DM incidence	Secondary	Noise	Self-report	Individual	Strong
Lee et al. [45]	2015	Korea	High	Cross-sectional	13,478	47.6 ± 12.2	T2DM/T1DM prevalence	Secondary	PA	GIS	Aggregate	Moderate
Liu et al. [79]	2014	US	High	Cross-sectional	17,254	46.5 ± 18.5	T2DM/T1DM prevalence	Blood sample	PA, food	Self-report	Individual	Strong
Loo et al. [62]	2017	Canada	High	Cross-sectional	78,023	35% 18–40 years 23% >65 years	Glycaemic marker: HbA1c and fasting glucose	Blood sample	PA	GIS	Individual	Strong
Maas et al. [66]	2009	Netherlands	High	Cross-sectional	345,103	38% >45 years 63% < 45 years	T2DM/T1DM prevalence	Secondary	PA	Register	Individual	Moderate
Mena et al. [53]	2015	Chile	High	Cross-sectional	832	45 ± 14	Glycaemic marker: Fasting glucose level	Blood sample	PA, food	GIS	Individual	Moderate
Meyer et al. [81]	2015	US	High	Longitudinal	14,379 (observations)	45.2 ± 3.6	Glycaemic marker: HOMA index	Blood sample	PA, food	GIS	Individual	Moderate
Mezuk et al. [70]	2016	Sweden	High	Longitudinal	2,948,851	NR	T2DM incidence	Secondary	Food	GIS	Individual	Strong
Morland et al. [75]	2006	US	High	Cross-sectional	10,763	100% >50 years	T2DM/T1DM prevalence	Blood sample	Food	GIS	Aggregate	Moderate
Müller-Riemenschneider et al. [65]	2013	Australia	High	Cross-sectional	5970	29% >65 years 30% < 45 years	T2DM prevalence	Self-report	PA	GIS	Individual	Strong
Myers et al. [63]	2016	US	High	Cross-sectional	NA	NR	T2DM/T1DM prevalence	Secondary	PA, food	Secondary	Aggregate	Moderate
Ngom et al. [64]	2016	Canada	High	Cross-sectional	3,920,000	NR	T2DM/T1DM prevalence	Secondary	PA	GIS	Aggregate	Strong
Paquet et al. [54]	2014	Australia	High	Longitudinal	3145	51.5 ± 15.5	T2DM incidence	Blood sample	PA, food	GIS	Individual	Moderate
Schootman et al. [56]	2007	US	High	Longitudinal	644	56.2 ± 4.3	T2DM/T1DM incidence	Self-report	PA, noise	Self-report, environmental audit	Individual	Moderate
Sørensen et al. [84]	2013	Denmark	High	Longitudinal	57,053	56.1 (50.7–64.2)	T2DM/T1DM incidence	Secondary	Noise	GIS	Individual	Moderate
Sundquist et al. [22]	2015	Sweden	High	Longitudinal	512,061	55 ± 14.9	T2DM incidence	Secondary	PA	GIS	Aggregate	Moderate

*GIS* geographic information systems, *NA* not applicable, *NR* not recorded, *PA* physical activity, *T1DM* type 1 diabetes mellitus, *T2DM* type 2 diabetes mellitus

^a^Prevalence is incidence or glycaemic marker level

^b^Blood sample: study diagnosed diabetes based on glycaemic marker or oral glucose tolerance test; secondary: from data sources such as national health survey; self-report: ever diagnosed with diabetes

Sixty studies compared T2DM risk/prevalence in urban vs. rural environments (Table 1 and Additional file 2). The studies rated weak (*n* = 16) did not differ in terms of country income levels from the other studies [25–40].

**Fig. 2:**
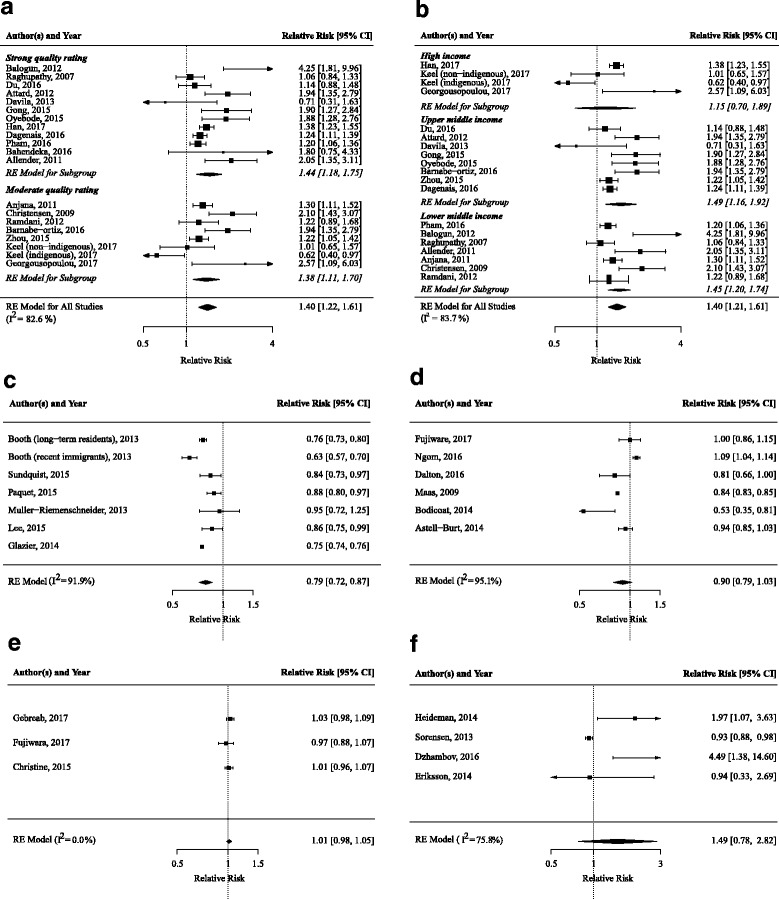
Forest plots of meta-analysis of the association between built environmental characteristics and T2DM risk/prevalence. **a** Urban vs. rural environments, stratified for study quality. **b** Urban vs. rural environments, stratified for country income level. **c** Walkability. **d** Green space. **e** Grocery stores. **f** Noise. T2DM type 2 diabetes mellitus. *RE model* random effects model

Of the remaining 44 studies, 25 (57%) of them found a higher risk or prevalence of T2DM in urban areas compared to rural areas. Altogether, 19 studies were eligible for the meta-analysis, which revealed a significantly higher risk/prevalence of T2DM in urban areas vs. rural areas (1.40; 95% CI, 1.22–1.61) (Fig. 2). This association was stronger in studies with strong quality ratings (1.44; 95% CI, 1.18–1.75), compared to those with moderate quality ratings (1.38; 95% CI, 1.11–1.70). After stratifying for country income level, one study was excluded [41] because the subgroup contained fewer than three studies. Associations were not different for upper-middle income countries (1.49; 95% CI, 1.16–1.92) and lower-middle income countries (1.45; 95% CI, 1.20–1.74), but were non-significant for high-income countries (1.16; 95% CI, 0.70–1.89).

Sensitivity analyses that included studies with weak quality ratings [33, 40] did not significantly change the results (Additional file 3).

Thirty studies investigated physical activity environment [19–22, 42–64] (Fig. 1, Table 2 and Additional file 2). All studies were performed in high-income level countries, except for one, which was performed in an upper-middle-level-income country [49].

Ten studies investigated the association between neighbourhood walkability and T2DM risk/prevalence. Six studies received a strong quality rating [20, 48, 57, 58, 62, 65]. Six studies observed that highly walkable neighbourhoods were associated with a lower T2DM risk/prevalence [19–22, 45, 54, 65]. In the meta-analyses of six studies, a pooled-risk ratio of 0.79 (95% CI, 0.72–0.87) was found, with an *I*^2^ for heterogeneity of 91.9%.

Six studies investigated the association between facilities for physical activity and T2DM risk/prevalence. Three studies received a strong quality rating [48, 49, 61]. Four studies did not observe an association between density of facilities and T2DM risk/prevalence [46, 48, 49, 61]. In two other studies, the higher availability of neighbourhood resources for physical activity was associated with lower T2DM risk [47, 63].

Seven studies investigated the association between green space and T2DM risk/prevalence. Two studies received a strong quality rating [44, 59]. Four studies observed that a higher availability of green space was associated with lower T2DM risk/prevalence [44, 54, 59, 64, 66]. One study observed that living closer to parks was significantly associated with higher prevalence of T2DM [64]. Aanother study observed a non-significant lower risk [42]. In meta-analyses of six studies, more green space tended to be associated with lower T2DM risk/prevalence with a pooled-risk ratio of 0.90 (95% CI, 0.79–1.03) with *I*^2^ for heterogeneity of 95.1%.

Four studies investigated infrastructure in relation to T2DM risk/prevalence. Two studies received a strong quality rating [49, 67]. Four studies did not observe an association between connectivity, infrastructure and road quality and T2DM risk/prevalence [49, 56, 68]. One study observed that a better transportation infrastructure, defined as more paved roads, was associated with higher T2DM prevalence [67].

Four studies investigated the association between safety and T2DM risk/prevalence. One study received a strong rating [49]. None of the studies showed an association between either traffic safety or safety from crime and T2DM risk/prevalence [49, 50, 56].

Twenty studies investigated characteristics of the food environment [46–48, 51–55, 60, 61, 63, 69–77] (Fig. 1, Table 2 and Additional file 2). All studies were performed in high-income-level countries.

Eight studies investigated the association between supermarkets and grocery stores and T2DM risk/prevalence. Two studies received a strong quality rating [61, 69]. One study observed that greater availability of grocery stores was associated with lower T2DM prevalence and that a higher percentage of households without a car located far from a supermarket was associated with higher T2DM prevalence [46]. A second study observed an unadjusted correlation between a greater distance to markets and lower fasting glucose levels [53]. Five studies did not observe a significant association between availability of supermarkets/grocery stores and T2DM prevalence [60, 61, 63, 69, 71, 75]. In a meta-analysis of three studies [48, 60, 61], a higher density of grocery stores was not associated with T2DM risk/prevalence (1.01; 95% CI, 0.98–1.05; *I*^2^ = 0%).

Seven studies investigated the association between availability of fast-food outlets and convenience stores and T2DM risk/prevalence. Three studies received a strong quality rating [61, 69, 72]. Four studies did not observe an association between availability of fast-food outlets/convenience stores and T2DM prevalence [61, 63, 69, 71, 75]. A higher availability of fast-food outlets and convenience stores was associated with higher T2DM prevalence in two studies [46, 72]. Studies could not be meta-analysed because the studies did not investigate consistent outcomes (T2DM risk vs. markers).

Four studies investigated the healthiness of the food environment subjectively or as an index and the association with T2DM risk/prevalence. One study received a strong quality rating [48]. Two studies focused on the perceived availability of healthy foods, rather than objectively measured availability. One study observed greater self-reported availability of healthy food resources to be associated with lower T2DM risk [47]. The second study assessed perceived availability, objective availability and a combination of the two, of which only perceived availability was associated with a lower T2DM risk [48]. Another study found no association between the presence of food deserts and T2DM prevalence [78].

Three studies used a ratio of unhealthful food stores to more healthful food stores, such as the Relative Food Environment Index (RFEI), with a higher value indicating an unhealthier food environment. One study received a strong quality rating [70]. This study observed that a higher ratio, i.e. a relatively unhealthier food environment, was associated with a higher risk of T2DM. Two studies did not observe consistent associations between RFEI and T2DM risk [54, 74].

Six studies used composite measures of physical activity and food-related built environmental characteristics (Tables 2 and 3, and Additional file 4). One study received a strong quality rating [79]. A summary score indicating the presence of more healthy food resources and physical activity resources was associated with lower T2DM incidence [47]. Furthermore, residing in a neighbourhood with physical and social-environmental disadvantages was associated with higher T2DM prevalence [79]. Clusters of large metropolitan counties, characterised by low population density, median income, low socioeconomic status index and greater access to food observed less T2DM [73]. Finally, no association was observed between vibrancy index, density and obesogenicity clusters and T2DM risk/prevalence [68, 80, 81].

**Table 3:** Study results of studies investigating physical activity environment, food environment, residential noise and diabetes mellitus

Author	Exposure	Study result	95% confidence interval or *p* value	Adjustment for confounding
Ahern et al., 2011 [46]	Food environment:	Beta (SE)		Age, obesity rate
	1. Percentage of households with no car living more than 1 mile from a grocery store	1. 0.07 (0.01)	1. *P* < 0.001	
	2. Fast-food restaurants per 1000	2. 0.41 (0.07)	2. *P* < 0.001	
	3. Full service restaurants per 1000	3. -0.15 (0.04)	3. *P* < 0.01	
	4. Grocery stores per 1000	4. -0.37 (0.09)	4. *P* < 0.001	
	5. Convenience stores per 1000	5. 0.30 (0.06)	5. *P* < 0.001	
	6. Direct money made from farm sales per capita	6. -0.01 (0.02)	6. *P* < 0.01	
	PA environment:			
	7. Recreational facilities per 1000	7. -0.12 (0.21)	7. NS	
AlHasan et al., 2016 [69]	Food outlet density:	Beta (SE)		Age, obesity, PA, recreation facility density, unemployed, education, household with no cars and limited access to stores, race
	1. Fast-food restaurant density per 1000 residents	1. -0.55 (0.90)	1. NS	
	2. Convenience store density	2. 0.89 (0.86)	2. NS	
	3. Super store density	3. -0.4 (11.66)	3. NS	
	4. Grocery store density	4. -3.7 (2.13)	4. NS	
Astell-Burt et al., 2014 [42]	Green space (percent):	OR:		Age, sex, couple status, family history, country of birth, language spoken at home, weight, psychological distress, smoking status, hypertension, diet, walking, MVPA, sitting, economic status, annual income, qualifications, neighbourhood affluence, geographic remoteness
	1. >81	1. 0.94	1. 0.85–1.03	
	2. 0–20	2. 1	2. NA	
Auchincloss et al., 2009 [47]	Neighbourhood resources:	HR:		Age, sex, family history, income, assets, education, ethnicity, alcohol, smoking, PA, diet, BMI
	1. Healthy food resources	1. 0.63	1. 0.42–0.93	
	2. PA resources	2. 0.71	2. 0.48–1.05	
	3. Summary score	3. 0.64	3. 0.44–0.95	
Bodicoat et al., 2014 [44]	Green space (percent)	OR:		Age, sex, area social deprivation score, urban/rural status, BMI, PA, fasting glucose, 2 h glucose, total cholesterol
	1. Least green space (Q1)	1. 1	1. NA	
	2. Most green space (Q4)	2. 0.53	2. 0.35–0.82	
Bodicoat et al., 2015 [72]		OR:		Age, sex, area social deprivation score, urban/rural status, ethnicity, PA
	1. Number of fast-food outlets (per 2)	1. 1.02	1. 1.00–1.04	
	2. Density of fast-food outlet (per 200 residents)	2. 13.84	2. 1.60–119.6	
Booth et al., 2013 [19]	Walkability:	HR:		Age, sex, income
	*Men*			
	*Recent immigrants*			
	1. Least walkable quintile	1. 1.58	1. 1.42–1.75	
	2. Most walkable quintile	2. 1	2. NA	
	*Long-term residents*			
	1. Least walkable quintile	1. 1.32	1. 1.26–1.38	
	2. Most walkable quintile	2. 1	2. NA	
	*Women*			
	*Recent immigrants*			
	1. Least walkable quintile	1. 1.67	1. 1.48–1.88	
	2. Most walkable quintile	2. 1	2. NA	
	*Long-term residents*			
	1. Least walkable quintile	1. 1.24	1. 1.18–1.31	
	2. Most walkable quintile	2. 1	2. NA	
Braun et al., 2016 [57, 58]	Walkability index, after residential relocation	Beta (SE)		1. Income, household size, marital status, employment status, smoking status, health problems that interfere with PA 2. Additionally, adjusted for age, sex, ethnicity, education
	1. Fixed-effects model	1. -0.011 (0.015)	1. *P* > 0.05	
	2. Random-effects model	2. -0.016 (0.010)	2. *P* > 0.05	
Braun et al., 2016 [57, 58]	Walkability: within person change in Street Smart Walk Score	Beta (SE): 0.999 (0.002)	*P* > 0.05	Age, sex, ethnicity, education, householdincome, employment status, marital status, neighbourhood SES
Cai et al., 2017 [82]	Daytime noise (dB)	Percentage change in fasting glucose per IQR Daytime noise: 0.2	95% CI, 0.1–0.3 *P* < 0.05	Age, sex, season of blood draw, smoking status and pack-years, education, employment, alcohol consumption, air pollution
Carroll et al., 2017 [71]	Count of fast-food outlets:	Beta per SD change: − 0.0094	-0.030–0.011	Age, sex, marital status, education, employment status, smoking status
	1. Interaction with overweight/obesity	1. −0.002	1. -0.023–0.019	
	2. Interaction with time	2. 0.0003	2. -0.003–0.004	
	3. Interaction with time and overweight/obesity	3. -0.002	3. -0.006–0.001	
	Count of healthful food resources:	0.012	-0.008–0.032	
	4. Interaction with overweight/obesity	4. 0.021	4. -0.000–0.042	
	5. Interaction with time	5. -0.003	5. -0.006–0.001	
	6. Interaction with time and overweight/obesity	6. -0.006	6. -0.009–-0.002	
Christine et al., 2015 [48]	Neighbourhood physical environment, diet related:	HR:		Age, sex, family history, household per capita income, educational level, smoking, alcohol, neighbourhood SES
	1. Density of supermarkets and/or fruit and vegetable markets (GIS)	1. 1.01	1. 0.96–1.07	
	2. Healthy food availability (self-report)	2. 0.88	2. 0.78–0.98	
	3. GIS and self-report combined measure	3. 0.93	3. 0.82–1.06	
	Neighbourhood physical environment, PA related:			
	1. Density of commercial recreational facilities (GIS)	1. 0.98	1. 0.94–1.03	
	2. Walking environment (self-report)	2. 0.80	2. 0.70–0.92	
	3. GIS and self-report combined measure	3. 0.81	3. 0.68–0.96	
Creatore et al., 2016 [20]	Walkability:	Absolute incidence rate difference over 12 years FU:		Age, sex, area income, ethnicity
	1. Low walkable neighbourhoods (Q1)	1. -0.65	1. -1.65–0.39	
	2. High walkable neighbourhoods over (Q5)	2. - 1.5	2. -2.6– -0.4	
Cunningham-Myrie et al., 2015 [49]	Neighbourhood characteristics:	OR:		Age, sex, district, fruit and vegetable intake
	1. Neighbourhood infrastructure	1. 1.02	1. 0.95–1.1	
	2. Neighbourhood disorder score	2. 0.99	2. 0.95–1.03	
	3. Home disorder score	3. 1	3. 0.96–1.03	
	4. Recreational space in walking distance	4. 1.12	4. 0.86–1.45	
	5. Recreational space availability	5. 1.01	5. 0.77–1.32	
	6. Perception of safety	6. 0.99	6. 0.88–1.11	
Dalton et al., 2016 [59]	Green space:	HR:		Age, sex, BMI, parental diabetes, SES Effect modification by urban-rural status and SES was investigated, but association was not moderated by either
	1. Least green space (Q1)	1. 1	1. NA	
	2. Most green space (Q4)	2. 0.81	2. 0.65–0.99	
	3. Mediation by PA	3. 0.96	3. 0.88–1.06	
Dzhambov et al., 2016 [83]	Day-evening-night equivalent sound level:	OR:		Age, sex, fine particulate matter, benzo alpha pyrene, BMI, family history of T2DM, subjective sleep disturbance, bedroom location
	1. 51–70 decibels	1. 1	1. NA	
	2. 71–80 decibels	2. 4.49	2. 1.39–14.7	
Eichinger et al., 2015 [50]	Characteristics of built residential environment:	Beta:		Age, sex, individual-level SES
	1. Perceived distance to local facilities	1. 0.006	1. *P* < 0.01	
	2. Perceived availability/maintenance of cycling/walking infrastructure	2. NS		
	3. Perceived connectivity	3. NS		
	4. Perceived safety with regards to traffic	4. NS		
	5. perceived safety from crime	5. NS		
	6. Neighbourhood as pleasant environment for walking/cycling	6. NS		
	7. Presence of trees along the streets	7. NS		
Eriksson et al., 2014 [85]	Aircraft noise level:	OR:		Age, sex, family history, SES based on education, PA, smoking, alcohol, annoyance due to noise
	1. <50 dB	1. 1	1. NA	
	2. ≥55 dB	2. 0.94	2. 0.33–2.70	
Flynt et al., 2015 [73]	Clusters (combination of number of counties, urban-rural classification, population density, income, SES, access to food stores, obesity rate, diabetes rate):	Median standardised diabetes mellitues rate:	IQR:	-
	1	1. 0	1. -0.05 - 0.7	
	2	2. 0	2. -0.04–0.7	
	3	3. 0	3. -0.08–0.01	
	4	4. -0.04	4. -1.01–0.6	
	5	5. -0.08	5. -1.5–-0.04 ANOVA: *p* < 0.001	
Frankenfeld et al., 2015 [74]	RFEI ≤ 1 clusters:	Predicted prevalence:		Demographic and SES variables
	1. Grocery stores	1. 7.1	1. 6.3–7.9	
	2. Restaurants	2. 5.9	2. 5.0–6.8, *p* < 0.01	
	3. Specialty foods	3. 6.1	3. 5.0–7.2, *p* < 0.01	
	RFEI >1:			
	4. Restaurants and fast-food	4. 6.0	4. 4.9–7.1, *p* < 0.01	
	5. Convenience stores	5. 6.1	5. 4.9–7.3, *p* < 0.01	
Freedman et al., 2011 [68]	Built environment:	OR:		Age, ethnicity, marital status, region of residence, smoking, education, income, childhood health, childhood SES, region of birth, neighbourhood scales
	*Men:*			
	1. Connectivity (2000 Topologically Integrated	1. 1.06	1. 0.86–1.29	
	Geographic Encoding and Referencing system)	2. 1.05	2. 0.89–1.24	
	2. Density (number of food stores, restaurants, housing units per square mile)			
	*Women:*			
	3. Connectivity	3. 1.01	3. 0.84–1.20	
	4. Density	4. 0.99	4. 0.99–1.17	
Fujiware et al., 2017 [60]	Count within neighbourhood unit (mean 6.31 ± 3.9 km^2^)	OR per IQR increase:		Age, sex, marital status, household number, income, working status, drinking, smoking, vegetable consumption, walking, going-out behaviour, frequency of meeting, BMI, depression
	1. Grocery stores	1. 0.97	1. 0.88–1.08	
	2. Parks	2. 1.16	2. 1–1.34	
Gebreab et al., 2017 [61]	Density within 1-mile buffer:	HR:		Age, sex, family history of diabetes, SES, smoking, alcohol consumption, physical activity, diet
	1. Favourable food stores	1. 1.03	1. 0.98–1.09	
	2. Unfavourable food stores	2. 1.07	2. 0.99–1.16	
	3. PA resources	3. 1.03	3. 0.98–1.09	
Glazier et al., 2014 [21]	Walkability index:	Rate ratio:		Age, sex
	1. Q1	1. 1	1. NA	
	2. Q5	2. 1.33	2. 1.33–1.33	
	Index components:			
	1. Population density (Q1: Q5)	1. 1.16	1. 1.16–1.16	
	2. Residential density (Q1: Q5)	2. 1.33	2. 1.33–1.33	
	3. Street connectivity (Q1: Q5)	3. 1.38	3. 1.38–1.38	
	4. Availability of walkable destinations (Q1: Q5)	4. 1.26	4. 1.26–1.26	
Heidemann et al., 2014 [86]	Residential traffic intensity:	OR:		Age, sex, smoking, passive smoking, heating of house, education, BMI, waist circumference, PA, family history
	1. No traffic	1. 1	1. NA	
	2. Extreme traffic	2. 1.97	2. 1.07–3.64	
Hipp et al., 2015 [78]	Food deserts	Correlation: NR	NS	–
Lee et al., 2015 [45]	Walkability:	OR:		Age, sex, smoking, alcohol, income level
	1. Community 1	1. 1	1. NA	
	2. Community 2	2. 0.86	2. 0.75–0.99	
Loo et al., 2017 [62]	Walkability (walk score) Difference between Q1 and Q4	Beta for HbA1C:		Age, sex, current smoking status, BMI, relevant medications and medical diagnoses, neighbourhood violent crime rates and neighbourhood indices of material deprivation, ethnic concentration, dependency, residential instability
		1. -0.06	1. -0.11–0.02	
		Beta for fasting glucose:		
		2. 0.03	2. -0.04–0.1	
Maas et al., 2009 [66]	Green space:	OR:		Demographic and socioeconomic characteristics, urbanicity
	1. Q1	1. 1	1. NA	
	2. Q4	2. 0.84	2. 0.83–0.85	
Mena et al., 2015 [53]		Correlation:		–
	1. Distance to parks	1. NR	1. NA	
	2. Distance to markets	2. -0.094	2. *P* < 0.05	
Mezuk et al., 2016 [70]	Ratio of the number of health-harming food outlets to the total number of food outlets within a 1000-m buffer of each person	OR per km^2^: 2.11	1.57–2.82	Age, sex, education, household income
Morland et al., 2006 [75]	Presence of:	Prevalence ratio:		Age, sex, income, education, ethnicity, food stores and service places, PA
	1. Supermarkets	1. 0.96	1. 0.84–1.1	
	2. Grocery stores	2. 1.11	2. 0.99–1.24	
	3. Convenience stores	3. 0.98	3. 0.86–1.12	
Müller-Riemenschneider et al., 2013 [65]	Walkability (1600 m buffer):	OR:		Age, sex, education, household income, marital status
	1. High walkability	1. 0.95	1. 0.72–1.25	
	2. Low walkability	2. 1	2. NA	
	Walkability (800 m buffer):			
	3. High walkability	3. 0.69	3. 0.62–0.90	
	4. Low walkability	4. 1	4. NA	
Myers et al., 2017 [63]	Physical activity:	Beta:		Age
	1. Recreation facilities per 1000	1. -0.457	1. -0.809– -0.104	
	2. Natural amenities (1–7)	2. 0.084	2. 0.042–0.127	
	Food:			
	3. Grocery stores and supercentres per 1000	3. 0.059	3. -0.09–0.208	
	4. Fast-food restaurants per 1000	4. -0.032	4. -0.125–0.062	
Ngom et al., 2016 [64]	Distance to green space:	Prevalence ratio:		Age, sex, social and environmental predictors
	1. Q1 (0–264 m)	1. 1	1. NA	
	2. Q4 (774–27781 m)	2. 1.09	2. 1.03–1.13	
Paquet et al., 2014 [54]	Built environment attributes:	RR:		Age, sex household income, education, duration of FU, area-level SES
	1. RFEI	1. 0.99	1. 0.9–1.09	
	2. Walkability	2. 0.88	2. 0.8–0.97	
	3. POS			
	a. POS count	a. 1	a. 0.92–1.08	
	b. POS size	b. 0.75	b. 0.69–0.83	
	c. POS greenness	c. 1.01	c. 0.9–1.13	
	d. POS type	d. 1.09	d. 0.97–1.22	
Schootman et al., 2007 [56]	Neighbourhood conditions (objective):	OR:		Age, sex, income, perceived income adequacy, education, marital status, employment, length of time at present address, own the home, area
	1. Housing conditions	1. 1.11	1. 0.63–1.95	
	2. Noise level from traffic, industry, etc.	2. 0.9	2. 0.48–1.67	
	3. Air quality	3. 1.2	3. 0.66–2.18	
	4. Street and road quality	4. 1.03	4. 0.56–1.91	
	5. Yard and sidewalk quality	5. 1.05	5. 0.59–1.88	
	Neighbourhood conditions (subjective):			
	6. Fair–poor rating of the neighbourhood	6. 1.04	6. 0.58–1.84	
	7. Mixed or terrible feeling about the neighbourhood	7. 1.1	7. 0.6–2.02	
	8. Undecided or not at all attached to the neighbourhood	8. 0.68	8. 0.4–1.18	
	9. Slightly unsafe–not at all safe in the neighbourhood	9. 0.61	9. 0.35–1.06	
Sørensen et al., 2013 [84]	Exposure to road traffic noise per 10 dB:	Incidence rate ratio:		Age, sex, education, municipality SES, smoking status, smoking intensity, smoking duration, environmental tobacco smoke, fruit intake, vegetable intake, saturated fat intake, alcohol, BMI, waist circumference, sports, walking, pollution
	1. At diagnosis	1. 1.08	1. 1.02–1.14	
	2. 5 years preceding diagnosis	2. 1.11	2. 1.05–1.18	
Sundquist et al., 2015 [22]	Walkability:	OR:		Age, sex, income, education, neighbourhood deprivation
	1. D1 (low)	1. 1.16	1. 1.00–1.34	
	2. D10 (high)	2. 1	2. NA	

*BMI* body mass index, *CI* Confidence interval, *GIS* graphical information system, *HR* hazard ratio, *IQR* interquartile range, *NA* not applicable, *NR* not reported, *NS* not significant, *OR* odds ratio, *PA* physical activity, *MVPA* moderate to vigorous physical activity, *POS* Public open space, *RFEI* Retail Food Environment Index, *RR* relative risk, *SD* standard deviation, *SE* standard error, *SES* socioeconomic status, *FU* follow-up

Four studies investigated the association between residential noise and T2DM risk/prevalence. One study received a strong quality rating [82]. All studies observed that higher exposure to residential noise was associated with increased T2DM risk/prevalence [82–85]. In meta-analyses of four studies [83–86], higher exposure to residential noise was not associated with T2DM risk/prevalence (1.49; 95% CI, 0.78–2.82, *I*^2^ = 75.8%).

## Discussion

This systematic review investigated evidence for the association between built environmental characteristics, related to lifestyle behaviours, and T2DM risk/prevalence, worldwide. The association between built environmental characteristics and T2DM risk/prevalence has been investigated a fair amount, with 84 studies on the subject, although for our review, 23 of these studies were excluded due to their low quality ratings. Urbanisation was associated with a higher T2DM risk/prevalence. The evidence for an association between the physical activity environment and T2DM risk was more consistent than it was for the food environment. Higher neighbourhood walkability was associated with lower T2DM risk and more green space tended to be associated with lower T2DM risk.

First, we observed that residing in urban areas was associated with higher T2DM risk/prevalence, in line with the findings of the IDF diabetes atlas [8] and a recent meta-analysis for South East Asia. Urbanisation is a process in which inhabitants of a particular region increasingly move to more densely populated areas. Urbanisation is a broad operationalisation of the built environment and includes a range of characteristics, such as higher availability of food, facilities, and infrastructure. In general, previous reviews have observed conflicting results for urbanisation [4, 5, 8]. Urbanisation has consistently been associated with less physical activity and unhealthier dietary habits, but also with higher total walking and cycling for transportation [4, 5, 8]. The observed heterogeneity in terms of results might be due to the variety of definitions used to classify an urban area, which is distinct for different countries and studies. To account for this, we stratified our analyses by country income level [18], and the majority of studies (38 out of 60) were conducted in middle-income countries, which reduces the heterogeneity in the studies included. It must be recognised that considerable heterogeneity in definitions of urban vs. rural exists beyond stratification on country income level. Across countries with the same country income level, there is large variety of what urban or rural areas may look like and the populations that reside in these areas. At present, there is no homogeneous and generally accepted definition of urban or rural areas and the majority of studies did not include a definition that was used to make this classification.

Second, the present study provides consistent evidence for an association between the built physical activity environment and T2DM risk/prevalence. Higher walkability and availability of green space were most consistently associated with lower T2DM risk/prevalence. Our results for urbanisation seem contradictory to the lower T2DM risk/prevalence associated with greater neighbourhood walkability, since greater walkability is often observed in more urbanised environments [5]. These seemingly contradictory results could be explained by the underrepresentation of high-income countries in the urban to rural comparison studies, and the overrepresentation of these countries in walkability studies. The (perceived) walkability of urban areas also varies across different parts of the world. So, whereas walkability may be a feature of cities in high-income regions, this may not be the case in cities in lower-income regions. Furthermore, urbanisation is a much broader construct than walkability, and even within one urban area, walkability may differ between or even within neighbourhoods. In addition, other urbanisation-related issues, besides walkability, may be more important, such as other physical activity environment characteristics and the food environment, which counterbalance the effects of walkability in urban areas. These results would suggest that certain aspects of the built food environment were associated with a higher T2DM risk, but we could not find consistent evidence of this in our review.

An association between the built food environment and T2DM risk/prevalence was not consistently observed. The availability of fast-food and convenience stores and the perceived healthiness of the food environment tended to be associated with higher T2DM risk/prevalence and lower T2DM risk/prevalence, respectively. However, due to heterogeneity in the studies, insufficient studies were available for meta-analysis, thus preventing us from drawing solid conclusions. The only possible meta-analyses were three studies including the density of grocery stores, but this confirmed that no significant associations could be observed. Also by reviewing the evidence, supermarkets and grocery stores and the RFEI were not associated with T2DM risk/prevalence. These findings are consistent with an earlier systematic review that reported that perceived availability was associated with healthy dietary behaviours [9], whereas objective measures of accessibility and availability of food environment yielded mixed results [9]. The association between the perceived environment and a healthier diet can be explained by not limiting the concept of environment to specific shops or locations, but rather to the participant’s resources for healthy food, e.g. gardens and markets. On the other hand, perceptions may also reflect an individual’s intentions and motivations rather than location alone. A difficulty with regard to establishing useful diet measures is that they are very heterogeneous and difficult to define. For instance, access to a supermarket is often seen as contributing to a healthy food environment, even though they are also sources of unhealthy products [9]. Establishing a comprehensive definition is further complicated because food can be bought in a variety of shops and locations that are not directly associated with food, e.g. at the counter of a pharmacy. The same heterogeneity was observed to a lesser extent in the built physical activity environment. For instance, infrastructure includes drivers for active transportation (sidewalks and cycling lanes) as well as for passive transportation (public transport and roads) [87]. We conclude that the heterogeneity in exposure assessment associated with built environmental variables made the examination of the associations with T2DM risk/prevalence more difficult.

Finally, although higher exposure to residential noise was consistently associated with higher T2DM risk/prevalence in individual studies, this was not confirmed in our meta-analysis, in contrast with an earlier meta-analysis [16]. This difference could be explained by the inclusion of only confounder adjusted risk ratios in our study.

A strength of this study is the comprehensive overview of the literature on the association between built environmental characteristics and T2DM risk/prevalence, in which we included worldwide evidence. We assessed study quality and took country income levels into account. However, certain limitations of this study need to be addressed.

A weakness of any systematic review and meta-analysis is that its quality is dependent on the quality of the studies included. For instance, not all studies that were included distinguished between T2DM and type 1 diabetes mellitus. However, the majority of all people with diabetes have T2DM so the evidence provided in our review was very likely applicable to T2DM risk/prevalence [1]. Secondly, because most studies in the present review were cross-sectional, our review cannot provide the foundation for causal inferences. Finally, publication bias could influence our findings, but our search turned out a relatively high number of null findings, suggesting publication bias an unlikely limitation. Finally, residential self-selection is an important issue that should be included in studies investigating the associations between built environment and disease. Self-selection occurs when residents choose a residence based on socioeconomic or other circumstances, or lifestyle preferences. Evidently, such selections may influence our results, as for instance higher socioeconomic status neighbourhoods may contain more green space, as well as more highly educated and health-conscious residents. However, the true effect of residential self-selection on these associations has often not been accounted for in the included studies and is difficult to investigate. One narrative review observed that studies using various approaches to identify self-selection (i.e. a questionnaire or statistical methods) explained only a minor part of the associations between built environment and travel behaviours [88]. Two studies included in the present review observed that residential relocation, as an indicator of self-selection, resulted in inconsistent effects on associations with health outcomes [57, 58]. It is, therefore, hard to conclude on the effect of self-selection bias on our results, based on the current evidence.

Despite the limitations of our study, our results may be relevant for infrastructure planning. For example, in addition to other positive consequences of walkability and access to green space, these environmental characteristics may also contribute to T2DM prevention. Future research should focus on developing a more homogeneous definition of environmental characteristics, particularly in relation to the food environment. Also, more in-depth explorations are necessary of the pathways through which environments affect diabetes risk, while taking the potential confounding variables into account.

## Conclusions

In conclusion, urbanisation is associated with higher T2DM risk/prevalence. The built physical activity environment - walkability and access to green space, in particular - was consistently associated with reduced T2DM risk/prevalence, while no consistent evidence was found for an association between the built food environment and T2DM risk/prevalence. These conclusions have implications in terms of urban planning and the inclusion of walkable and green cities.

## Additional files

Additional file 1: Search strategy (DOCX 21 kb)

Additional file 2: Study characteristics and results of studies with a weak quality rating (DOCX 43 kb)

Additional file 3: Sensitivity analyses (ZIP 120 kb)

Additional file 4: Study characteristics and results of studies investigating combination environmental characteristics. (DOCX 21 kb)
