# Correction to: Built environmental characteristics and diabetes: a systematic review and meta-analysis

**DOI:** 10.1186/s12916-020-01882-6

**Published:** 2021-03-07

**Authors:** N. R. den Braver, J. Lakerveld, F. Rutters, L. J. Schoonmade, J. Brug, J. W. J. Beulens

**Affiliations:** 1 Department of Epidemiology & Biostatistics, Amsterdam Public Health, Research Institute, VU University Medical Center, De Boelelaan 1089a, 1081HV Amsterdam, The Netherlands; 2 University Library, VU, Amsterdam, The Netherlands; 3 Amsterdam School for Communication Research, University of Amsterdam, Amsterdam, The Netherlands; 4 Julius Center for Health Sciences and Primary Care, University Medical Center Utrecht, Utrecht, The Netherlands

**Correction to: BMC Med (2018) 16:12**

**https://doi.org/10.1186/s12916-017-0997-z**

After publication, it came to the authors’ attention that after revision and update of the literature search, some numbers were inconsistently implemented (differences between tables and text) and some reference categories were incorrectly transformed in the original article [1]. This Correction displays the corrected information ahead. These adjustments did not change the results.

- The third sentence of the Findings sub-section of the Abstract should instead state the following:

Higher neighbourhood walkability was associated with lower T2DM risk/prevalence (n=6, OR=0.79 (95%-CI=0.7-0.9; I2=92%)) and more green space was associated with lower T2DM risk/prevalence (n=4, OR=0.91 (95%-CI=0.88–0.95; I2=0%)).

- The eighth paragraph of the Results should instead state the following:

Eight studies investigated the association between green space and T2DM risk/prevalence. Two studies received a strong quality rating [44, 59]. Five studies observed that a higher availability of green space was associated with lower T2DM risk/prevalence [44, 54, 59, 64, 66] and three studies did not observe an association [42, 53, 60]. In meta-analyses of four studies, more green space was associated with lower T2D risk/prevalence with a pooled-risk ratio of 0.91(95%-CI: 0.88 – 0.95) with an I2 for heterogeneity of 0%.

- The final paragraph of the Results should instead state the following:

Five studies investigated the association between residential noise and T2DM risk/prevalence. One study received a strong quality rating [82]. Four studies observed that higher exposure to residential noise was associated with increased T2D risk/prevalence [82-85], and two studies did not observe an association [56, 85]. In meta-analyses of three studies [83, 85, 86], higher exposure to residential noise was not associated with T2DM risk/ prevalence (1.95 (95%CI: 0.96 – 3.97), I2 = 44.2%).

- Figure 1 has been amended and the correct version can be viewed ahead.

**Fig. 1:**
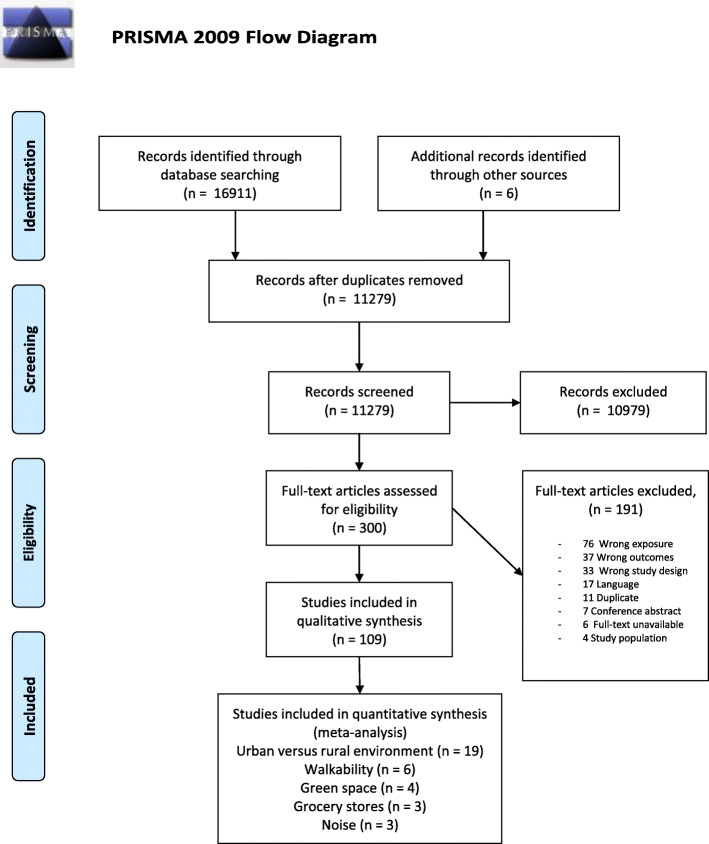
Flow chart of study inclusion

- Figure 2 has been amended and the correct version can be viewed ahead along with its corrected caption.

**Fig. 2:**
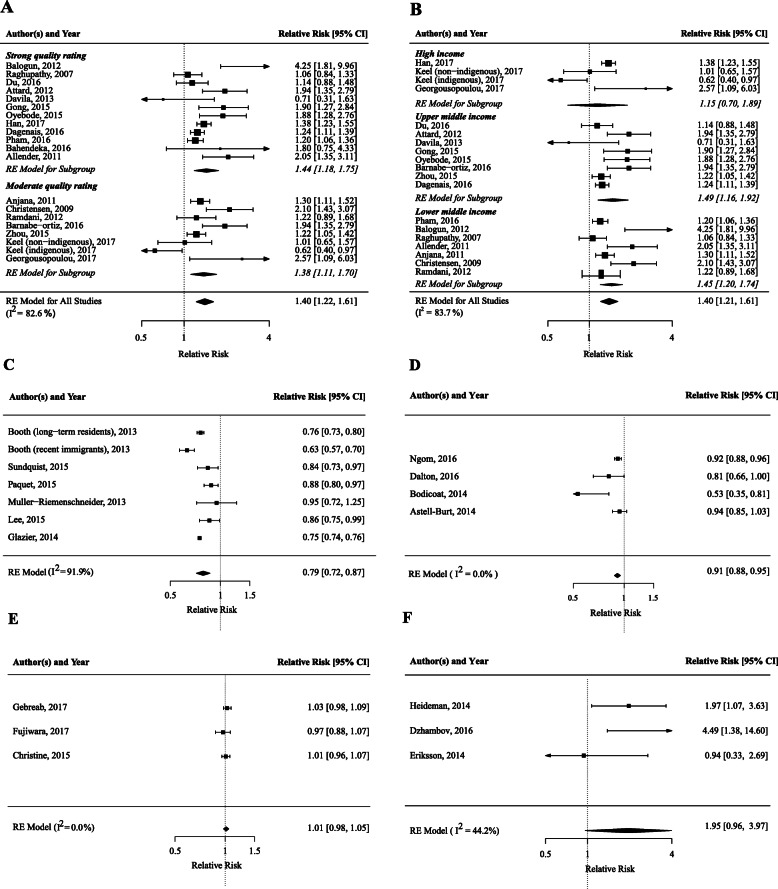
Forest plots of meta-analysis of the association between built environmental characteristics and T2DM risk/prevalence. **a** urban versus rural environments, stratified for study quality; **b** urban versus rural environments, stratified for country income level; **c** walkability; **d** green space, **e** grocery stores, **f** noise

- The heading of Table 3 should instead state the following:

**Table 3**: Study results of studies investigating the association of physical activity environment, food environment or residential noise with T2D.

- The caption of Supplementary Table 2 should instead state the following:

**Table 3:** Study results of studies investigating the association of physical activity environment, food environment or residential noise with T2D

Author	Exposure	Study result	95% Confidence interval or *p*-value	Adjustment for confounding
Ahern et al., 2011	Food environment:	Beta (SE)		Age and obesity rate
	1. % household with no car living more than 1 mile from a grocery store	1. 0.07 (0.01)	1. *P* < 0.001	
	2. fast food restaurants per 1000	2. 0.41 (0.07)	2. *P* < 0.001	
	3. Full service restaurants per 1000	3. -0.15 (0.04)	3. *P* < 0.01	
	4. grocery stores per 1000	4. -0.37 (0.09)	4. *P* < 0.001	
	5. convenience stores per 1000	5. 0.30 (0.06)	5. *P* < 0.001	
	6. direct money made from farm sales per capita	6. -0.01 (0.02)	6. *P* < 0.01	
	PA environment:			
	7. recreational facilities per 1000	7. -0.12 (0.21)	7. NS	
AlHasan et al., 2016	Food outlet density:	Beta (SE)		Age, obesity, PA, recreation facility density, unemployed, education, household with no cars and limited access to store and race.
	1. Fast food restaurant density (per 1000 residents)	1. -0.55 (0.90)	1. NS	
	2. Convenience store density	2. 0.89 (0.86)	2. NS	
	3. Super store density	3. -0.4 (11.66)	3. NS	
	4. Grocery store density	4. -3.7 (2.13)	4. NS	
Astell-Burt et al., 2014	Green space (percent):	OR:	95%CI:	age, sex, couple status, family history, country of birth, language spoken at home, weight, psychological distress, smoking status, hypertension, diet, walking, MVPA, sitting, economic status, annual income, qualifications, neighbourhood affluence, geographic remoteness.
	1. >81	1. 0.94	1. 0.85 - 1.03	
	2. 0-20	2. 1	2. NA	
Auchincloss et al., 2009	Neighbourhood resources:	HR:	95%CI:	Age, sex, family history, income, assets, education, ethnicity, alcohol, smoking, PA, diet, BMI
	1. Healthy food resources	1. 0.63	1. 0.42 – 0.93	
	2. PA resources	2. 0.71	2. 0.48 – 1.05	
	3. Summary score	3. 0.64	3. 0.44 – 0.95	
Bodicoat et al., 2014	Green space (percent)	OR:	95%CI:	Age, sex, area social deprivation score, urban/rural status, BMI, PA, fasting glucose, 2 h glucose, total cholesterol
	1. Least green space (Q1)	1. 1	1. NA	
	2. Most green space (Q4)	2. 0.53	2. 0.35 – 0.82	
Bodicoat et al., 2015		OR:	95%CI:	Age, sex, area social deprivation score, urban/rural status, ethnicity, PA
	1. Number of fast-food outlets (per 2)	1. 1.02	1. 1.00 – 1.04	
	2. Density of fast-food outlet (per 200 residents)	2. 13.84	2. 1.60 – 119.6	
Booth et al., 2013	Walkability:	HR:	95%CI:	Age, sex, income
	Men	Men		
	Recent immigrants	Recent immigrants		
	1. Least walkable quintile	1. 1.58	1. 1.42 – 1.75	
	2. Most walkable quintile	2. 1	2. NA	
	Long-term residents	Long-term residents:		
	1. Least walkable quintile	1. 1.32	1. 1.26 – 1.38	
	2. Most walkable quintile	2. 1	2. NA	
	Women	Women		
	Recent immigrants	Recent immigrants:		
	1. Least walkable quintile	1. 1.67	1. 1.48 – 1.88	
	2. Most walkable quintile	2. 1	2. NA	
	Long-term residents	Long –term residents:		
	1. Least walkable quintile	1. 1.24	1. 1.18 – 1.31	
	2. Most walkable quintile	2. 1	2. NA	
Braun et al., 2016	Walkability index, after residential relocation	Beta (SE)		
	1. Fixed effects model	1. -0.011 (0.015)	1. *P* > 0.05	1. income, household size, marital status, employment status, smoking status, health problems that interfere with PA
	2. Random effects model	2. -0.016 (0.010)	2. *P* > 0.05	2. Additionally adjusted for age, gender, ethnicity, education
Braun et al., 2016	Walkability: within person change in Street Smart Walk Score	Beta (SE): 0.999 (0.002)	*P* > 0.05	Age, sex, ethnicity, education, household income, employment status, marital status, neighbourhood SES
Cai et al., 2017	Daytime noise (dB)	% change in fasting glucose per IQR daytime noise: 0.2	95%CI: 0.1 – 0.3 *P* < 0.05	age, sex, season of blood draw, smoking status and pack-years, education, employment and alcohol consumption, air pollution
Carroll et al., 2017		Beta per SD change:	95% CI:	Age, sex, marital status, education, employment status, and smoking status
	Count of fast-food outlets:	−0.0094	-0.030 – 0.011	
	1. Interaction with overweight/obesity	1. −0.002	1. -0.023 – 0.019	
	2. Interaction with time	2. 0.0003	2. -0.003 – 0.004	
	3. Interaction with time and overweight/obesity	3. -0.002	3. -0.006 – 0.001	
	Count of healthful food resources:	0.012	-0.008 – 0.032	
	4. Interaction with overweight/obesity	4. 0.021	4. -0.000 – 0.042	
	5. Interaction with time	5. -0.003	5. -0.006 – 0.001	
	6. Interaction with time and overweight/obesity	6. -0.006	6. -0.009 – -0.002	
Christine et al., 2015	Neighbourhood physical environment, diet related:	HR:	95%CI:	Age, sex, family history, household per capita income, educational level, smoking, alcohol, neighbourhood SES
	1. Density of supermarkets and/or fruit and vegetable markets (GIS)	1. 1.01	1. 0.96 – 1.07	
	2. Healthy food availability (self-report)	2. 0.88	2. 0.78 – 0.98	
	3. GIS and self-report combined measure	3. 0.93	3. 0.82 – 1.06	
	Neighbourhood physical environment, PA related:			
	1. Density of commercial recreational facilities (GIS)	1. 0.98	1. 0.94 – 1.03	
	2. Walking environment (self-report)	2. 0.80	2. 0.70 – 0.92	
	3. GIS and self-report combined measure	3. 0.81	3. 0.68 – 0.96	
Creatore et al., 2016	Walkability:	Absolute incidence rate difference over 12 year FU:	95%CI:	Age, sex, area income and ethnicity
	1. Low walkable neighbourhoods (Q1)	1. -0.65	1. -1.65 – 0.39	
	2. High walkable neighbourhoods over (Q5)	2. - 1.5	2. -2.6 – -0.4	
Cunningham-Myrie et al, 2015	Neighbourhood characteristics:	OR:	95%CI:	Age, sex, district, fruit and vegetable intake
	1. Neighbourhood infrastructure	1. 1.02	1. 0.95 – 1.1	
	2. Neighbourhood disorder score	2. 0.99	2. 0.95 – 1.03	
	3. Home disorder score	3. 1	3. 0.96 – 1.03	
	4. Recreational space in walking distance	4. 1.12	4. 0.86 – 1.45	
	5. Recreational space availability	5. 1.01	5. 0.77 – 1.32	
	6. Perception of safety	6. 0.99	6. 0.88 – 1.11	
Dalton et al., 2016	Green space:	HR:	95%CI:	Age, sex, BMI, parental diabetes, and SES. Effect modification by urban-rural status and SES was investigated, but association was not moderated by either
	1. Least green space (Q1)	1. 1	1. NA	
	2. Most green space (Q4)	2. 0.81	2. 0.65 – 0.99	
	3. Mediation by PA	3. 0.96	3. 0.88 -1.06	
Dzhambov et al., 2016	Day-evening-night equivalent sound level:	OR:	95%:	Age, sex, fine particulate matter, benzo alpha pyrene, body mass index, family history of T2D, subjective sleep disturbance, and bedroom location
	1. 51-70 decibels	1. 1	1. NA	
	2. 71-80 decibels	2. 4.49	2. 1.39 – 14.7	
Eichinger et al., 2015	Characteristics of built residential environment:	Beta:		Age, sex, individual-level SES
	1. Perceived distance to local facilities	1. 0.006	P < 0.01	
	2. Perceived availability / maintenance of cycling/walking infrastructure	2. NS		
	3. Perceived connectivity	3. NS		
	4. Perceived safety with regards to traffic	4. NS		
	5. perceived safety from crime	5. NS		
	6. Neighbourhood as pleasant environment for walking / cycling	6. NS		
	7. Presence of trees along the streets	7. NS		
Eriksson et al., 2014	Aircraft noise level:	OR:	95%CI:	Age, sex, family history, SES based on education, PA, smoking, alcohol, annoyance due to noise.
	1. <50 dB	1. 1	1. NA	
	2. ≥55 dB	2. 0.94	2. 0.33 – 2.70	
Flynt et al., 2015	Clusters (combination of number of counties, urban-rural classification, population density, income, SES, access to food stores , obesity rate, diabetes rate):	Median standardized DM rate:	IQR:	-
	1	1. 0	1. -0.05 - 0.7	
	2	2. 0	2. -0.04 – 0.7	
	3	3. 0	3. -0.08 – 0.01	
	4	4. -0.04	4. -1.01 – 0.6	
	5	5. -0.08	5. -1.5 – -0.04	
			ANOVA: p < 0.001	
Frankenfeld et al., 2015	RFEI^†^ ≤ 1 Clusters:	Predicted prevalence:	95%CI:	Demographic and SES variables
	1. Grocery stores	1. 7.1	1. 6.3 – 7.9	
	2. Restaurants	2. 5.9	2. 5.0 – 6.8, p < 0.01	
	3. Specialty foods	3. 6.1	3. 5.0 – 7.2, p < 0.01	
	RFEI^†^ > 1:			
	4. Restaurants and fast food	4. 6.0	4. 4.9 – 7.1, p < 0.01	
	5. Convenience stores	5. 6.1	5. 4.9 – 7.3, p < 0.01	
Freedman et al., 2011	Built environment:	OR:	95%CI:	Age, ethnicity, marital status, region of residence, smoking, education, income, childhood health, childhood SES, region of birth, neighbourhood scales
	Men:			
	1. Connectivity (2000 Topologically Integrated Geographic Encoding and Referencing system).	1. 1.06	1. 0.86 – 1.29	
	2. Density (number of food stores, restaurants, housing units per square mile)	2. 1.05	2. 0.89 – 1.24	
	Women:			
	3. Connectivity	3. 1.01	3. 0.84 – 1.20	
	4. Densityx	4. 0.99	4. 0.99 – 1.17	
Fujiware et al., 2017	Count within neighbourhood unit (mean 6.31 ± 3.9 km^2^)	OR per IQR increase:	95%CI:	age, sex, marital status, household number, income, working status, drinking, smoking, vegetable consumption, walking, going-out behaviour, frequency of meeting, BMI, depression
	1. Grocery stores	1. 0.97	1. 0.88 – 1.08	
	2. Parks	2. 1.15	2. 0.98 – 1.34	
Gebreab et al., 2017	Density within 1 mile buffer:	HR:	95%CI:	age, sex, family history of diabetes, SES, smoking, alcohol consumption, PA and diet
	1. Favourable food stores	1. 1.03	1. 0.98 – 1.09	
	2. Unfavourable food stores	2. 1.07	2. 0.99 – 1.16	
	3. PA resources	3. 1.03	3. 0.98 – 1.09	
Glazier et al., 2014	Walkability index:	Rate ratio:	95%CI:	Age and sex
	1. Q1	1. 1	1. NA	
	2. Q5	2. 1.33	2. 1.33 – 1.33	
	Index components:			
	1. Population density (Q1: Q5)	1. 1.16	1. 1.16 – 1.16	
	2. Residential density (Q1: Q5)	2. 1.33	2. 1.33 – 1.33	
	3. Street connectivity (Q1: Q5)	3. 1.38	3. 1.38 – 1.38	
	4. Availability of walkable destinations (Q1: Q5)	4. 1.26	4. 1.26 – 1.26	
Heidemann et al., 2014	Residential traffic intensity:	OR:	95%CI:	Age, sex, smoking, passive smoking, heating of house, education, BMI, waist circumference, PA, family history
	1. No traffic	1. 1	1. NA	
	2. Extreme traffic	2. 1.97	2. 1.07 – 3.64	
Hipp et al., 2015	Food deserts	Correlation: NR	NS	-
Lee et al., 2015	Walkability:	OR:	95%CI:	Age, sex, smoking, alcohol, income level
	1. Community 1	1. 1	1. NA	
	2. Community 2	2. 0.86	2. 0.75 – 0.99	
Loo et al., 2017	Walkability (Walk score) Difference between Q1 and Q4	Beta for HbA1C:		Age, sex, current smoking status, BMI, relevant medications and medical diagnoses, neighbourhood violent crime rates and neighbourhood indices of material deprivation, ethnic concentration, dependency and residential instability
		1. -0.06	1. -0.11 – 0.02	
		Beta for fasting glucose:		
		2. 0.03	2. -0.04 – 0.1	
Maas et al., 2009	Green space: per 10% more green space in 1 km radius	OR: 0.98	95%CI: 0.97 – 0.99	Demographic and socioeconomic characteristics, urbanity
Mena et al., 2015		Correlation:		-
	1. Distance to parks	1. NR	1. NA	
	2. Distance to markets	2. -0,094	2. *P* < 0.05	
Mezuk et al., 2016	Ratio of the number of health-harming food outlets to the total number of food outlets within a 1,000-m buffer of each person	OR: 2.11	95%CI: 1.57 – 2.82	Age, sex, education, and household income
Morland et al., 2006	Presence of:	Prevalence ratio:	95%CI:	Age, sex, income, education, ethnicity, food stores and service places,, PA
	1. Supermarkets	1. 0.96	1. 0.84 – 1.1	
	2. Grocery stores	2. 1.11	2. 0.99 – 1.24	
	3. Convenience stores	3. 0.98	3. 0.86 – 1.12	
Müller-Riemenschneider et al., 2013	Walkability (1,600 m buffer):	OR:	95%CI:	Age, sex, education, household income, marital status.
	1. High walkability	1. 0.95	1. 0.72 – 1.25	
	2. Low walkability	2. 1	2. NA	
	Walkability (800 m buffer):			
	3. High walkability	3. 0.69	3. 0.62 – 0.90	
	4. Low walkability	4. 1	4. NA	
Myers et al., 2016	Physical activity:	Beta:	95%CI:	Age
	1. Recreation facilities per 1000	1. -0.457	1. -0.809 – -0.104	
	2. Natural amenities (1 – 7)	2. 0.084	2. 0.042 – 0.127	
	Food:			
	3. Grocery stores & supercentres per 1000	3. 0.059	3. -0.09 – 0.208	
	4. Fast food restaurants per 1000	4. -0.032	4. -0.125 – 0.062	
Ngom et al., 2016	Distance to green space:	PR:	95%CI:	Age, sex, social and environmental predictors
	1. Q1 (0 – 264 m)	1. 1	1. NA	
	2. Q4 (774 – 27781 m)	2. 1.09	2. 1.03 – 1.13	
Paquet et al., 2014	Built environmental attributes:	RR:	95%CI:	Age, sex household income, education, duration of FU, area-level SES.
	1. RFEI^¥^	1. 0.99	1. 0.9 – 1.09	
	2. Walkability	2. 0.88	2. 0.8 – 0.97	
	3. POS			
	a. POS count	a. 1	a. 0.92 – 1.08	
	b. POS size	b. 0.75	b. 0.69 – 0.83	
	c. POS greenness	c. 1.01	c. 0.9 – 1.13	
	d. POS type	d. 1.09	d. 0.97 – 1.22	
Schootman et al., 2007	Neighbourhood conditions (objective):	OR:	95%CI:	Age, sex, income, perceived income adequacy, education, marital status, employment, length of time at present address, own the home, area
	1. Housing conditions	1. 1.11	1. 0.63 – 1.95	
	2. Noise level from traffic, industry, etc.	2. 0.9	2. 0.48 – 1.67	
	3. Air quality	3. 1.2	3. 0.66 – 2.18	
	4. Street and road quality	4. 1.03	4. 0.56 – 1.91	
	5. Yard and sidewalk quality	5. 1.05	5. 0.59 – 1.88	
	Neighbourhood conditions (subjective):			
	6. Fair - poor rating of the neighbourhood	6. 1.04	6. 0.58 – 1.84	
	7. Mixed or terrible feeling about the neighbourhood	7. 1.1	7. 0.6 – 2.02	
	8. Undecided or not at all attached to the neighbourhood	8. 0.68	8. 0.4 – 1.18	
	9. Slightly unsafe - not at all safe in the neighbourhood	9. 0.61	9. 0.35 – 1.06	
Sørensen et al., 2013	Exposure to road traffic noise per 10 dB:	Incidence rate ratio:	95%CI:	Age, sex, education, municipality SES, smoking status, smoking intensity, smoking duration, environmental tobacco smoke, fruit intake, vegetable intake, saturated fat intake, alcohol, BMI, waist circumference, sports, walking, pollution.
	1. At diagnosis	1. 1.08	1. 1.02 – 1.14	
	2. 5 years preceding diagnosis	2. 1.11	2. 1.05 – 1.18	
Sundquist et al., 2015	Walkability:	OR:	95%CI:	Age, sex, income, education, neighbourhood deprivation.
	1. D1 (low)	1. 1.16	1. 1.00 – 1.34	
	2. D10 (high)	2. 1	2. NA	

*Abbreviations*: *NA* not applicable, *NS* not significant, *NR* not reported, *95%CI* 95% Confidence interval, *RFEI* Retail Food Environment Index, *PSE* Neighbourhood physical and social environment, *POS* Public open space, *SE* standard error, *RR* relative risk, *OR* odds ratio, *HR* hazard ratios

*Prevalence; Beta (SE); RR; OR; HR, quality of accessible groceries, likelihood that neighbours help each other, examples of neighbours working together, sense of belonging, degree of trust in neighbours, poverty level

† RFEI = ratio of fast-food restaurants and unhealthful food stores to healthful food stores

**Additional File 2**: Study characteristics and results of studies with a weak quality rating

- The corrected version of Supplementary Table 2 can be viewed attached alongside this Correction article (as ‘Additional file 1’ here).

## Supplementary Information

**Additional file 1.**
