# Supplementary material for: Built environmental characteristics and diabetes: a systematic review and meta-analysis
Source: BMC Med. 2018 Jan 31;16:12. doi: 10.1186/s12916-017-0997-z (PMC5791730; doi:10.1186/s12916-017-0997-z)
Supplement: Supplementary file 1 — Search strategy (DOCX 21 kb) [file 12916_2017_997_MOESM1_ESM.docx]

**Additional file 1: Search strategy**

Search strategy in PubMed June 6^th^, 2017 (read from bottom-up).

| **Set** | **Search terms** | **Result** |
| --- | --- | --- |
| #3 | #1 AND #2 | 4199 |
| #2 | "Environment Design"[Mesh] OR "City Planning"[Mesh] OR "Spatial Analysis"[Mesh] OR "Geographic Information Systems"[Mesh] OR "Noise"[Mesh] OR "Parks, Recreational"[Mesh] OR "Crowding"[Mesh] OR green space*[tiab] OR greenspace*[tiab] OR green environment*[tiab] OR green infrastructure*[tiab] OR natural space*[tiab] OR natural environment*[tiab] OR natural infrastructure*[tiab] OR environment design[tiab] OR environmental influence*[tiab] OR environmental determinant*[tiab] OR environmental support*[tiab] OR environmental approach*[tiab] OR environmental variable*[tiab] OR environmental attribute*[tiab] OR environmental barrier*[tiab] OR environmental characteristic*[tiab] OR environmental correlat*[tiab] OR environment design*[tiab] OR city planning*[tiab] OR urban design[tiab] OR urban planning*[tiab] OR urban form[tiab] OR town planning*[tiab] OR neighbourhood*[tiab] OR neighborhood*[tiab] OR geospatial[tiab] OR local environment*[tiab] OR rural environment*[tiab] OR urban environment*[tiab] OR objective environment*[tiab] OR perceived environment*[tiab] OR measured environment*[tiab] OR obesogenic environment*[tiab] OR built environment*[tiab] OR physical environment*[tiab] OR geoepidemiology[tiab] OR spatial analysis[tiab] OR land use[tiab] OR spatial access[tiab] OR residential environment*[tiab] OR urban-rural epidemiology[tiab] OR geographic cluster*[tiab] OR residential factor*[tiab] OR residence characteristic*[tiab] OR geographic information system*[tiab] OR geographical information system*[tiab] OR sprawl[tiab] OR zoning[tiab] OR residential location*[tiab] OR residential proximit*[tiab] OR population densit*[tiab] OR food outlet*[tiab] OR grocery store*[tiab] OR fast food density[tiab] OR fast food restaurant*[tiab] OR retail densit*[tiab] OR walkability[tiab] OR cyclability[tiab] OR sidewalk*[tiab] OR pedestrian[tiab] OR cycle path*[tiab] OR cyclepath*[tiab] OR recreational facilit*[tiab] OR recreation facility*[tiab] OR worksite*[tiab] OR sports facilit*[tiab] OR food environment*[tiab] OR food suppl*[tiab] OR public open space*[tiab] OR crowding[tiab] OR park access[tiab] OR urban park*[tiab] OR noise pollution[tiab] OR contextual research[tiab] OR ecological stud*[tiab] OR ecological analys*[tiab] OR remoteness[tiab] OR aesthetic*[tiab] OR active travel*[tiab] OR passive travel*[tiab] OR travel to work[tiab] | 176994 |
| #1 | "Diabetes Mellitus"[Mesh:NoExp] OR "Diabetes Mellitus, Type 2"[Mesh] OR "Blood Glucose"[Mesh] OR "Hyperglycemia"[Mesh] OR "Insulin Resistance"[Mesh] OR diabetes[tiab] OR diabetic*[tiab] OR dm2[tiab] OR niddm[tiab] OR dm 2[tiab] OR T2DM*[tiab] OR dm type 2[tiab] OR insulin*[tiab] OR glucose[tiab] OR prediabetes[tiab] OR pre-diabetes[tiab] OR prediabetic[tiab] OR glycemic[tiab] OR glycaemic[tiab] OR glycemia*[tiab] OR glycaemia*[tiab] OR HbA1[tiab] OR HB A1[tiab] OR HbA1c[tiab] OR hemoglobin*[tiab] OR haemoglobin*[tiab] OR hyperglycemia*[tiab] | 1099161 |

Search strategy in Embase.com June 6^th^, 2017 (read from bottom-up).

| **Set** | **Search terms** | **Result** |
| --- | --- | --- |
| #4 | #3 NOT ('conference abstract'/it OR 'editorial'/it OR 'erratum'/it OR 'letter'/it OR 'note'/it) | 8,047 |
| #3 | #1 AND #2 | 10,243 |
| #2 | 'environmental planning'/exp OR 'city planning'/exp OR 'spatial analysis'/exp OR 'geographic information system'/exp OR 'noise pollution'/exp OR 'land use'/exp OR ‘neighborhood'/exp OR 'recreational park'/exp OR 'crowding (area)'/exp OR ‘green space*‘:ab,ti OR greenspace*:ab,ti OR ‘green environment*‘:ab,ti OR ‘green infrastructure*’:ab,ti OR ‘natural space*’:ab,ti OR ‘natural environment*’:ab,ti OR ‘natural infrastructure*’:ab,ti OR ‘environment* design*’:ab,ti OR ‘environment* influence*’:ab,ti OR ‘environment* determinant*’:ab,ti OR ‘environment* support*’:ab,ti OR ‘environment* approach*’:ab,ti OR ‘environment* variable*’:ab,ti OR ‘environment* attribute*’:ab,ti OR ‘environment* barrier*’:ab,ti OR ‘environment* characteristic*’:ab,ti OR ‘environment* correlat*’:ab,ti OR ‘city planning*’:ab,ti OR ‘urban design*’:ab,ti OR ‘urban form*’:ab,ti OR ‘urban planning*’:ab,ti OR ‘town planning*’:ab,ti OR neighbourhood*:ab,ti OR neighborhood*:ab,ti OR geospatial:ab,ti OR ‘local environment*’:ab,ti OR ‘rural environment*’:ab,ti OR ‘urban environment*’:ab,ti OR ‘objective environment*’:ab,ti OR ‘perceived environment*’:ab,ti OR ‘measured environment*’:ab,ti OR ‘obesogenic environment*’:ab,ti OR ‘built environment*’:ab,ti OR ‘physical environment*’:ab,ti OR geoepidemiology:ab,ti OR ‘spatial analysis’:ab,ti OR ‘land use’:ab,ti OR ‘spatial access’:ab,ti OR ‘residential environment*’:ab,ti OR ‘urban rural epidemiology ‘:ab,ti OR ‘geographic cluster*’:ab,ti OR ‘residential factor*’:ab,ti OR ‘residence characteristic*’:ab,ti OR ‘geographic* information system*’:ab,ti OR sprawl:ab,ti OR zoning:ab,ti OR ‘residential location*’:ab,ti OR ‘resident* proximit*’:ab,ti OR ‘population densit*’:ab,ti OR ‘food outlet*’:ab,ti OR ‘grocery store*’:ab,ti OR ‘fast food densit*’:ab,ti OR ‘fast food restaurant*’:ab,ti OR ‘retail densit*’:ab,ti OR walkability:ab,ti OR cyclability:ab,ti OR sidewalk*:ab,ti OR pedestrian:ab,ti OR ‘cycle path*’:ab,ti OR cyclepath*:ab,ti OR ‘recreation* facilit*’:ab,ti OR worksite*:ab,ti OR ‘sport* facilit*’:ab,ti OR ‘food environment*’:ab,ti OR ‘food suppl*’:ab,ti OR ‘public open space*’:ab,ti OR crowding:ab,ti OR ‘park access’:ab,ti OR ‘urban park*’:ab,ti OR ‘noise pollution’:ab,ti OR ‘contextual research’:ab,ti OR ‘ecological stud*’:ab,ti OR ‘ecological analys*’:ab,ti OR remoteness:ab,ti OR aesthetic*:ab,ti OR ‘active travel*’:ab,ti OR ‘passive travel*’:ab,ti OR ‘travel to work’:ab,ti | 279,152 |
| #1 | 'diabetes mellitus'/de OR 'non insulin dependent diabetes mellitus'/exp OR 'glucose blood level'/exp OR 'hyperglycemia'/exp OR 'insulin resistance'/exp OR 'insulin sensitivity'/exp OR diabetes:ab,ti OR diabetic*:ab,ti OR dm2:ab,ti OR niddm:ab,ti OR ‘dm 2’:ab,ti OR T2DM*:ab,ti OR ‘dm type 2’:ab,ti OR insulin*:ab,ti OR glucose:ab,ti OR prediabetes:ab,ti OR ‘pre-diabetes’:ab,ti OR prediabetic:ab,ti OR glycemic:ab,ti OR glycaemic:ab,ti OR glycemia*:ab,ti OR glycaemia*:ab,ti OR HbA1:ab,ti OR ‘HB A1’:ab,ti OR HbA1c:ab,ti OR hemoglobin*:ab,ti OR haemoglobin*:ab,ti OR hyperglycemia*:ab,ti | 1,558,105 |

Search strategy in Web of Science Core Collection, June 6^th^, 2017 (read from bottom-up).

| **Set** | **Search terms** | **Result** |
| --- | --- | --- |
| #4 | #2 AND #1  **Refined by:** **WEB OF SCIENCE CATEGORIES:** ( ENDOCRINOLOGY METABOLISM OR PUBLIC ENVIRONMENTAL OCCUPATIONAL HEALTH OR GERIATRICS GERONTOLOGY OR SPORT SCIENCES OR NUTRITION DIETETICS OR ECOLOGY OR HEALTH POLICY SERVICES OR MEDICINE GENERAL INTERNAL OR BEHAVIORAL SCIENCES OR ENVIRONMENTAL SCIENCES OR MULTIDISCIPLINARY SCIENCES OR PATHOLOGY OR FOOD SCIENCE TECHNOLOGY OR PHYSIOLOGY OR GEOSCIENCES MULTIDISCIPLINARY OR CARDIAC CARDIOVASCULAR SYSTEMS OR HEALTH CARE SCIENCES SERVICES OR PSYCHOLOGY OR ANTHROPOLOGY )  *Indexes=SCI-EXPANDED, SSCI, A&HCI, ESCI Timespan=All years* | 4,665 |
| #3 | #1 AND #2 | 12,711 |
| #2 | TS=("environment design*" OR "city planning*" OR "spatial analysis" OR "geographic information system*" OR noise OR crowding OR “green space*” OR greenspace* OR "green environment*" OR "green infrastructure*" OR "natural space*" OR "natural environment*" OR "natural infrastructure*" OR "environmental influence*" OR "environmental determinant*" OR "environmental support*" OR "environmental approach*" OR "environmental variable*" OR "environmental attribute*" OR "environmental barrier*" OR "environmental characteristic*" OR "environmental correlat*" OR "urban design*" OR "urban planning*" OR "urban form" OR "town planning*" OR neighbourhood* OR neighborhood* OR geospatial OR "local environment*" OR "rural environment*" OR "urban environment*" OR "objective environment*" OR "perceived environment*" OR "measured environment*" OR "obesogenic environment*" OR "built environment*" OR "physical environment*" OR geoepidemiology OR "spatial analysis" OR "land use" OR "spatial access" OR "residential environment*" OR "urban-rural epidemiology" OR "geographic cluster*" OR "residential factor*" OR "residence characteristic*" OR "geographic information system*" OR "geographical information system*" OR sprawl OR zoning OR "residential location*" OR "residential proximit*" OR "population densit*" OR "food outlet*" OR "grocery store*" OR "fast food density" OR "fast food restaurant*" OR "retail densit*" OR walkability OR cyclability OR sidewalk* OR pedestrian OR "cycle path*" OR cyclepath* OR "recreational facilit*" OR "recreational park*" OR "recreation facility*" OR worksite* OR "sports facilit*" OR "food environment*" OR "food suppl*" OR "public open space*" OR crowding OR "park access" OR "urban park*" OR "noise pollution" OR "contextual research" OR "ecological stud*" OR "ecological analys*" OR remoteness OR aesthetic* OR "active travel*" OR "passive travel*" OR "travel to work") *Indexes=SCI-EXPANDED, SSCI, A&HCI, ESCI Timespan=All years* | 1,298,838 |
| #1 | TS=(diabetes OR diabetic* OR "blood glucose" OR "hyperglycemia" OR insulin* OR dm2 OR niddm OR dm 2 OR T2DM* OR dm type 2 OR glucose OR prediabetes OR “pre-diabetes” OR prediabetic OR glycemic OR glycaemic OR glycemia* OR glycaemia* OR HbA1 OR “HB A1” OR HbA1c OR hemoglobin* OR haemoglobin* OR hyperglycemia*) *Indexes=SCI-EXPANDED, SSCI, A&HCI, ESCI Timespan=All years* | 1,280,762 |
