# Supplementary material for: Built environmental characteristics and diabetes: a systematic review and meta-analysis
Source: BMC Med. 2018 Jan 31;16:12. doi: 10.1186/s12916-017-0997-z (PMC5791730; doi:10.1186/s12916-017-0997-z)
Supplement: Supplementary file 2 — Study characteristics and results of studies with a weak quality rating (DOCX 43 kb) [file 12916_2017_997_MOESM2_ESM.docx]

**Additional file 2: Study characteristics and results of studies with a weak quality rating**

**Supplementary table 1: study characteristics and results of studies with a weak quality rating investigating the association between urban – rural environment and DM in.**

| Author | Year | Country | Country income level | Study design | Sample size | Age | Outcome† | Outcome assessment‡ | Result: | | | Adjustment for Confounding | Quality statement |  |
| --- | --- | --- | --- | --- | --- | --- | --- | --- | --- | --- | --- | --- | --- | --- |
|  |  |  |  |  |  |  |  |  | *Urban > rural* | *Rural > urban* | *No difference* |  |  |  |
| Asadollahi et al. | 2015 | Iran | Upper middle | Cross-sectional | 2,158 |  | T2DM/T1DM prevalence | Blood sample | X |  |  | - | Weak |  |
| Azizi et al. | 2003 | Iran | Upper middle | Cross-sectional | 595,717 |  | T2DM/T1DM prevalence | Blood sample | X |  |  | - | Weak |  |
| Bharati et al. | 2011 | India | Lower middle | Cross-sectional | 214 |  | T2DM/T1DM prevalence | Self-report | X |  |  | Family history, WH ratio | Weak |  |
| Ceesay et al. | 1997 | Sierra Leone | Low | Cross-sectional | 501 |  | Glycaemic marker: random blood glucose | Blood sample | X |  |  | - | Weak |  |
| Colleran et al. | 2007 | Mexico | Upper middle | Cross-sectional | 200 |  | T2DM/T1DM prevalence | Secondary |  |  | X | - | Weak |  |
| Dar et al. | 2015 | India | Lower middle | Cross-sectional | 3,972 |  | T2DM prevalence | Blood sample | X |  |  | - | Weak |  |
| Gangqiang et al. | 2004 | China | Upper middle | Longitudinal | 3,650,000 |  | T2DM/T1DM incidence | Secondary |  |  | X | - | Weak |  |
| Khan et al. | 2014 | Bangladesh | Lower middle | Cross-sectional | 3,720 |  | T2DM/T1DM prevalence | Secondary | X |  |  | Region, age, education, marrital status, owning a TV, land ownership. Statified for sex. | Weak |  |
| Kodaman et al. | 2016 | Ghana | Lower middle | Cross-sectional | 3,316 | 43.5 ± 13.4 | T2DM/T1DM prevalence | Blood sample | X |  |  | Sex | Weak |  |
| Mi et al. | 2016 | China | Upper middle | Cross-sectional | 231,289 | 56.4 ± 11.4 | T2DM/T1DM prevalence | Blood sample | X |  |  | Age and sex | Weak |  |
| Mierzecki et al. | 2014 | Poland | High | Cross-sectional | 271 |  | Glycaemic marker: fasting blood glucose | Blood sample | X |  |  | Age | Weak |  |
| Mohamud et al. | 2010 | Malaysia |  | Cross-sectional | 4341 | 47.8 ± 14.5 | Insulin resistance: HOMA-IR ≥ 2.6 | Blood sample |  |  | X | No | Weak |  |
| Nakibuuka et al. | 2015 | Uganda | Low | Cross-sectional | 5,420 |  | T2DM/T1DM prevalence | Blood sample |  |  | X | - | Weak |  |
| Njelekela et al. | 2003 | Tanzania | Low | Cross-sectional |  |  | Glycaemic marker: HbA1c | Blood sample | X (women) |  | X (men) | Age | Weak |  |
| Shera et al. | 2007 | Pakistan | Lower middle | Cross-sectional | 5,433 |  | T2DM/T1DM prevalence | Secondary |  |  | X | - | Weak |  |
| Valverde et al. | 2006 | Spain | High | Cross-sectional | 1,556 |  | T2DM/T1DM prevalence | Blood sample |  |  | X | - | Weak |  |

**Supplementary table 2: study characteristics of studies with a weak quality rating investigating physical activity environment, food environment, residential noise and DM.**

| Author | Year | Country | Income level | Study design | Sample size | Outcome† | Outcome assessment‡ | Exposure category | Exposure assessment | Level geodata | Quality statement |
| --- | --- | --- | --- | --- | --- | --- | --- | --- | --- | --- | --- |
| Babey et al. ^65^ | 2008 | US | High | Cross-sectional |  | T2DM/T1DM prevalence rate | Self-report | Food | GIS | Individual | Weak |
| Ewing et al. ^103^ | 2014 | US | High | Cross-sectional | 709,234 | T2DM/T1DM prevalence | Blood sample | PA | Secondary | Aggregate | Weak |
|  |  |  |  |  |  |  |  |  |  |  |  |
| Herrick et al. ^50^ | 2015 | US | High | Cross-sectional | 15,522 | T2DM/T1DM prevalence | Blood sample | PA, food | Place of residence | Individual | Weak |
| Jiao et al. ^66^ | 2015 | US | High | Cross-sectional | 2,001 | T2DM/T1DM prevalence | Blood sample | Food | GIS | Individual | Weak |
| Marshall et al. ^51^ | 2014 | US | High | Cross-sectional | 1,044 | T2DM/T1DM prevalence | Self-report | PA, food | GIS, environmental audit | Aggregate | Weak |
| Salois et al. ^54^ | 2012 | US | High | Cross-sectional | NA | T2DM/T1DM prevalence | Secondary | PA, food | Secondary | Aggregate | Weak |
| Shaffer et al. | 2017 | US | High | Cross-sectional | 21.3 ± 1.3 | Glycaemix marker: fasting glucose | Blood sample | PA | Self-report | Individual | Weak |

**Supplementary table 3: study results of studies a weak quality rating investigating physical activity environment, food environment, residential noise and DM.**

| Author | Exposure | Study result* | 95% Confidence interval or p-value | At least age and sex adjusted |
| --- | --- | --- | --- | --- |
| Babey et al., 2008 | Food environment: RFEI^¥^   1. RFEI > 5 2. RFEI 3 - 4,9 3. RFEI < 3 | Prevalence:   1. 8.1% 2. 7.8% 3. 6.6% | P < 0.05 (high vs. low RFEI) | - |
| Ewing et al., 2014 | 1. Original sprawl index (density) 2. Refined sprawl index‡ | T-ratio   1. -2.22 2. -2.27 | 1. P < 0,05 2. P < 0,05 | Age, sex, ethnicity, income, education. |
| Herrick et al., 2015 | 1. Walkability (per SD change) 2. Supermarket density (per square mile) | OR:   1. 1.19 2. 0.84 | 95%CI:   1. 1.04 – 1.37 2. 0.71 – 0.99 | Age, sex, BMI, non-HDL cholesterol, SBP |
|  |  |  |  |  |
| Jiao et al., 2015 | Distance to closest fast food restaurant | OR: 1.29 | 95%CI: 0.83 – 1.99 | Age, sex, ethnicity, children under 12, children between 12-18, household size, income, employment |
| Marshall et al., 2014 | Block group level variables   1. Connectivity variables 2. Intersection density (per square mile) 3. Number of fast food restaurants 4. Number of big box stores 5. Number of grocery stores   City level:   1. Intersection density (per square mile) 2. Average tot number of lanes on major streets 3. Percent of major streets with bike lanes 4. Number of fast food restaurants 5. Number of fitness centres 6. Number of convenience stores | Beta (SE):   1. NR 2. NR 3. NR 4. 0.014 (SE NR) 5. NR 6. -0.0004 7. 0.029 8. -0.07 9. -0.001 10. NR 11. 0.008 | 1. NS 2. NS 3. NS 4. P < 0.10 5. NS 6. P < 0.05 7. P < 0.05 8. P < 0.05 9. P < 0.05 10. NS 11. P < 0.05 | - |
| Salois et al., 2012 | Local food economy:   1. Farmers' market density 2. Direct farm sales per capita (dollars) 3. Percent of farms with direct sales 4. Fast food restaurants density 5. Full-service restaurants density 6. Supermarkets-grocery store density 7. Convenience stores no gas density 8. Convenience stores with gas density 9. Supercenters and club density 10. Recreational and fitness facilites density 11. ERS natural amanitiy index | Intercept = 9.5. beta:   1. -0.925 2. -0.013 3. -0.007 4. 0.321 5. -0.606 6. -0.002 7. 1.993 8. 0.199 9. 1.69 10. -0.644 11. -0.051 | 1. P < 0.05 2. P < 0.01 3. NS 4. P < 0.01 5. P < 0.01 6. NS 7. P < 0.01 8. NS 9. NS 10. NS 11. NS | Age |
| Shaffer et al., 2017 | Walkability:  Males:   1. sidewalks 2. traffic 3. crime during day 4. crime at night   Females   1. sidewalks 2. traffic 3. crime during day 4. crime at night | Correlation:   1. 0.17 2. -0.08 3. -0.09 4. -0.02 5. 0.09 6. 0.28 7. 0.21 8. -0.16 | 1. P > 0.05 2. P > 0.05 3. P > 0.05 4. P > 0.05 5. P > 0.05 6. P < 0.05 7. P < 0.05 8. P > 0.05 | - |
