# Supplementary figures and images for: Built environmental characteristics and diabetes: a systematic review and meta-analysis
Source: BMC Med. 2018 Jan 31;16:12. doi: 10.1186/s12916-017-0997-z (PMC5791730; doi:10.1186/s12916-017-0997-z)

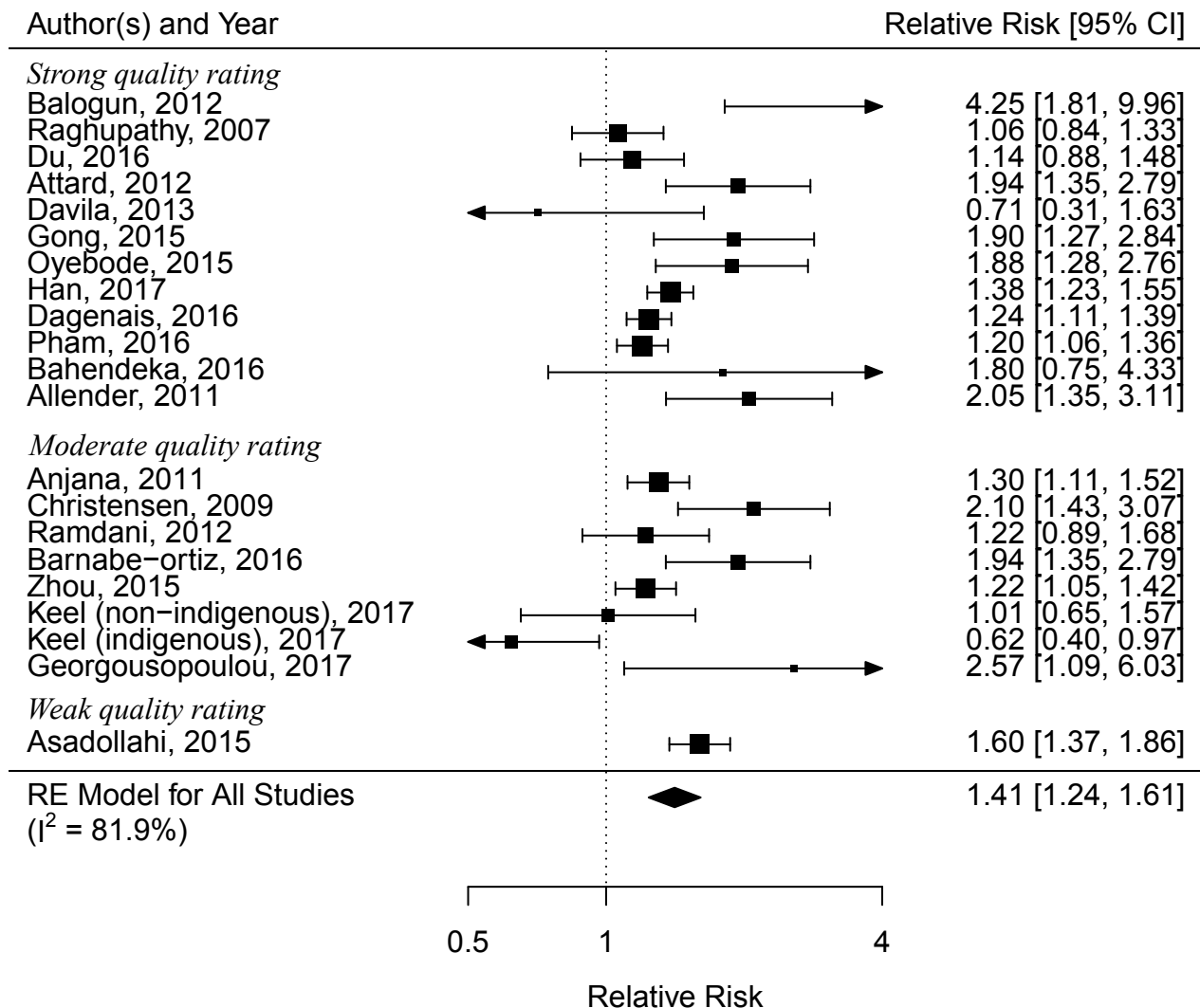

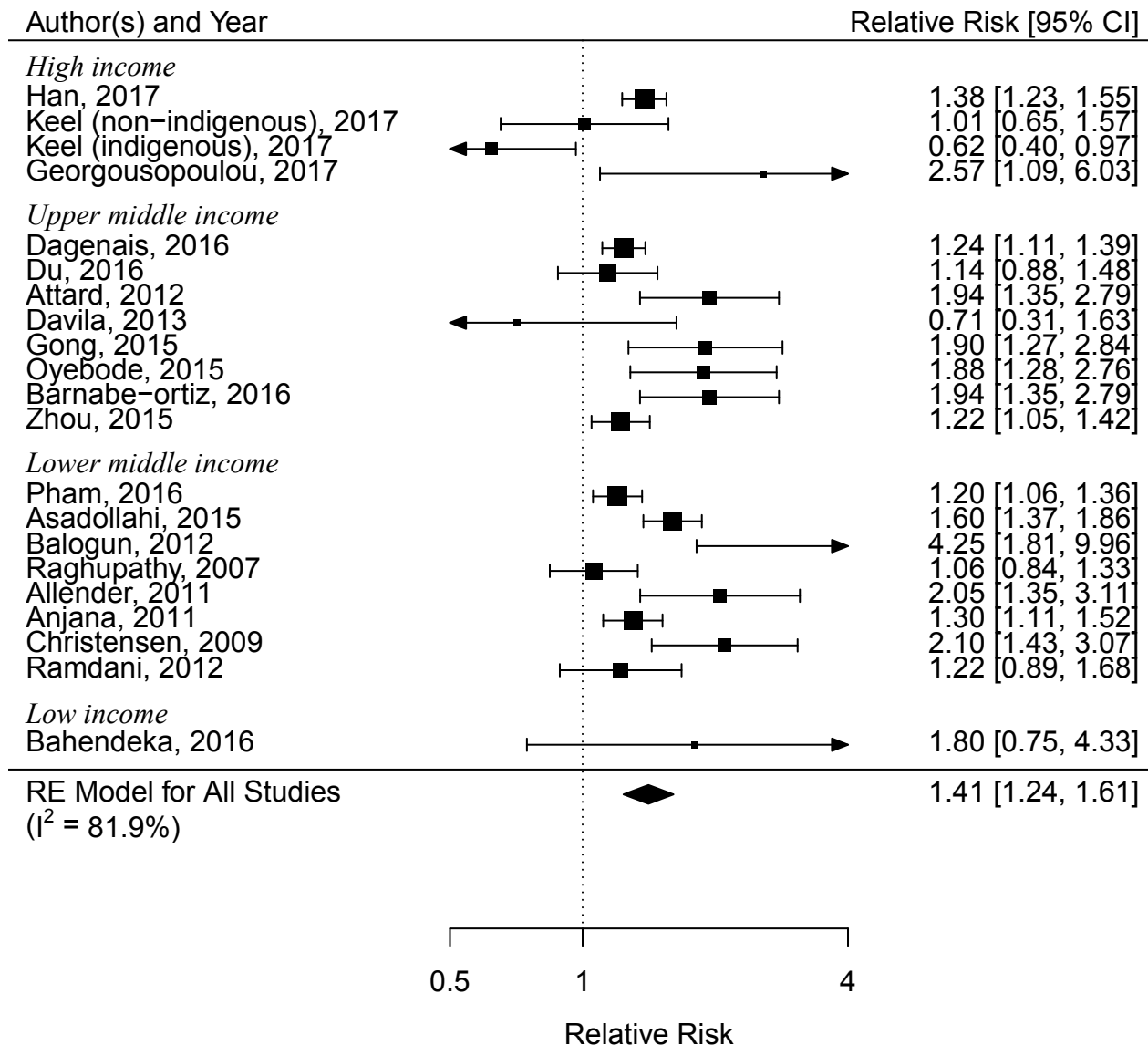

Supplement: Supplementary file 3 — Sensitivity analyses (ZIP 120 kb) [file 12916_2017_997_MOESM3_ESM.zip › 12916_2017_997_MOESM3_ESM/Additional file 3.1 Forest urban-rural sensitivity weakR1.pdf]

Author(s) and Year

Relative Risk [95% CI]

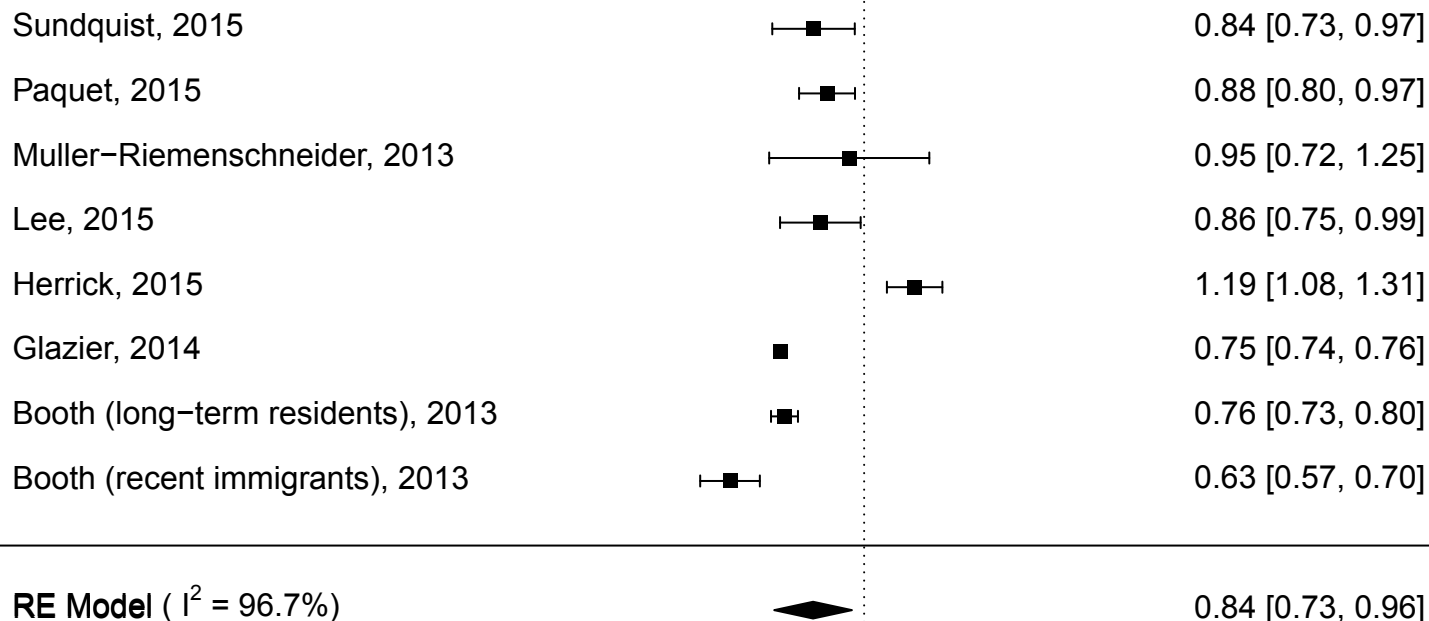

0.5 1 1.5

Relative Risk

Supplement: Supplementary file 3 — Sensitivity analyses (ZIP 120 kb) [file 12916_2017_997_MOESM3_ESM.zip › 12916_2017_997_MOESM3_ESM/Additional file 3.2 Forest walkability sensitivity weakR1.pdf]
