# Supplementary material for: Built environmental characteristics and diabetes: a systematic review and meta-analysis
Source: BMC Med. 2018 Jan 31;16:12. doi: 10.1186/s12916-017-0997-z (PMC5791730; doi:10.1186/s12916-017-0997-z)
Supplement: Supplementary file 4 — Study characteristics and results of studies investigating combination environmental characteristics. (DOCX 21 kb) [file 12916_2017_997_MOESM4_ESM.docx]

**Additional file 4: Study characteristics and results of studies investigating combination environmental characteristics.**

**Supplementary table 4: Studies investigating combination environmental characteristics.**

| Author | Exposure | Study result* | 95% Confidence interval or p-value | Adjustment for confounding |
| --- | --- | --- | --- | --- |
| Braun et al., 2015 | Vibrancy index† | Beta (SE): -0.002 (0.001) | NS | Demographic, socioeconomic and economic covariates |
| Freedman et al., 2011 | Built environment:  Men:   1. Connectivity (2000 Topologically Integrated Geographic Encoding and Referencing system). 2. Density (number of food stores, restaurants, housing units per square mile)   Women:   1. Connectivity 2. Density | OR:   1. 1.06 2. 1.05 3. 1.01 4. 0.99 | 95%CI:   1. 0.86 – 1.29 2. 0.89 – 1.24 3. 0.84 – 1.20 4. 0.99 – 1.17 | Age, ethnicity, marital status, region of residence, smoking status, years of education completed, mean assets, income category, childhood health, childhood SES, region of birth, neighbourhood scales. |
|  |  |  |  |  |
| Liu et al., 2014 | PSE ^Δ^:   1. Q1 ( < 0.62) 2. Q4 (0.70 – 0.76) | OR:   1. 1 2. 1.53 | 95%CI:   1. NA 2. 1.25 – 1.88 | Age, sex, ethnicity, education level, smoking status, body weight, physical activity, vegetable and/or fruit intake, study period |
| Meyer et al., 2015 | Obesogenicity clusters (latent class analyses)  Lower population density clusters:   1. Low obesogenicity, moderate devolpment: moderate level of neighbourhood features, relatively more Pa resources (tan the other two low-population-density classes) and a diverse mix of food resources. 2. Moderate obesogenicity, moderate development: high connectivity, moderate Pa resources, high convenience store, supermarkets, grocery store (relative to other food resources). 3. High obesogenicity, low development: low connectivity, few PA resources, food environment relatively high in convenience sotre and moderat in fast food.   Higher population density clusters:   1. Low obesogenicity, high development: high connectivity, many PA resources, food environment characterized by high in coops, specialty markest, and non-fastfood restaurants, low in convenience stores. 2. Moderate obesogenicity, moderate development: moderate levels of all features. 3. High obesogenicity, high development: high connectivity, large number of parks and PA resources, convenience stores, grocery stores. | Beta:   1. 0 2. -0.038 3. -0.013 4. 0 5. 0.034 6. -0.007 | 95%CI:   1. NA 2. -0.083 – 0.006 3. -0.045 – 0.019 4. NA 5. 0.002 – 0.067 6. -0.045 – 0.032 | Age, study year, race, sex, study center, educational attainment, census tract-level education, income. |

†Vribrancy index = composed of compactness, density, regional connectivity, local connectivity, destination accessibility, mixed use, social diversity, ‡ Refined sprawl index = density, land use mix, population and employment centering, street accessibility, Δ PSE = access to and usage of recreational facilities, access to fruits and vegetables, quality of accessible groceries, likelihood that neighbors help each other, examples of neighbors working together, sense of belonging, degree of trust in neighbors, poverty level.
