# Supplementary material for: Correction to: Built environmental characteristics and diabetes: a systematic review and meta-analysis
Source: BMC Med. 2021 Mar 7;19:63. doi: 10.1186/s12916-020-01882-6 (PMC7937232; doi:10.1186/s12916-020-01882-6)
Supplement: Supplementary file 1 — Additional file 1. [file 12916_2020_1882_MOESM1_ESM.docx]

Supplementary File 2: Study characteristics and results of studies with a weak quality rating

Supplementary Table 2.1: study characteristics and results of studies with a weak quality rating investigating the association of urban and rural environment with T2D

| Author | Year | Country | Country income level | Study design | Sample size | Age | Outcome† | Outcome assessment‡ | Result: | | | At least adjusted for age and sex | Quality statement |  |
| --- | --- | --- | --- | --- | --- | --- | --- | --- | --- | --- | --- | --- | --- | --- |
|  |  |  |  |  |  |  |  |  | *Urban > rural* | *Rural > urban* | *No difference* |  |  |  |
| Asadollahi et al. | 2015 | Iran | Upper middle | Cross-sectional | 2,158 | 45.5 ± 14.0 | T2D/T1D prevalence | Blood sample | X |  |  | Yes | Weak |  |
| Azizi et al. | 2003 | Iran | Upper middle | Cross-sectional | 595,717 | 62% < 50y  38% ≥ 50y | T2D/T1D prevalence | Blood sample | X |  |  | No | Weak |  |
| Bharati et al. | 2011 | India | Lower middle | Cross-sectional | 214 | 100% > 60y | T2D/T1D prevalence | Self-report | X |  |  | No | Weak |  |
| Ceesay et al. | 1997 | Sierra Leone | Low | Cross-sectional | 501 | 35.7 ± 15.7 | Glycaemic marker: random blood glucose | Blood sample | X |  |  | No | Weak |  |
| Colleran et al. | 2007 | Mexico | Upper middle | Cross-sectional | 200 | 100% > 50 years | T2D/T1D prevalence | Secondary |  |  | X | No | Weak |  |
| Dar et al. | 2015 | India | Lower middle | Cross-sectional | 3,972 | 100% > 40y | T2D prevalence | Blood sample | X |  |  | No | Weak |  |
| Gangqiang et al. | 2004 | China | Upper middle | Longitudinal | 3,650,000 | NA | T2D/T1D incidence | Secondary |  |  | X | No | Weak |  |
| Khan et al. | 2014 | Bangladesh | Lower middle | Cross-sectional | 7,543 | 100% > 35 y | T2D/T1D prevalence | Secondary | X |  |  | Yes | Weak |  |
| Kodaman et al. | 2016 | Ghana | Lower middle | Cross-sectional | 3,316 | 43.5 ± 13.4 | T2D/T1D prevalence | Blood sample | X |  |  | Sex | Weak |  |
| Mi et al. | 2016 | China | Upper middle | Cross-sectional | 231,289 | 56.4 ± 11.4 | T2D/T1D prevalence | Blood sample | X |  |  | Age and sex | Weak |  |
| Mierzecki et al. | 2014 | Poland | High | Cross-sectional | 271 | 25-45 years | Glycaemic marker: fasting blood glucose | Blood sample | X |  |  | No | Weak |  |
| Mohamud et al. | 2010 | Malaysia |  | Cross-sectional | 4341 | 47.8 ± 14.5 | Insulin resistance: HOMA-IR ≥ 2.6 | Blood sample |  |  | X | No | Weak |  |
| Nakibuuka et al. | 2015 | Uganda | Low | Cross-sectional | 5,420 | Urban:  32.7 ± 12.4  Rural:  40.1 ±16.3 | T2D/T1D prevalence | Blood sample |  |  | X | No | Weak |  |
| Njelekela et al. | 2003 | Tanzania | Low | Cross-sectional | 445 | 51.8 ± 3.4 | Glycaemic marker: HbA1c | Blood sample | X (women) |  | X (men) | No | Weak |  |
| Shera et al. | 2007 | Pakistan | Lower middle | Cross-sectional | 5,433 | 74% < 55y  26% ≥ 55y | T2D/T1D prevalence | Secondary |  |  | X | No | Weak |  |
| Valverde et al. | 2006 | Spain | High | Cross-sectional | 1,556 | 58% < 50y  42% ≥ 50y | T2D/T1D prevalence | Blood sample |  |  | X | No | Weak |  |

Supplementary Table 2.2:Study characteristics of studies with a weak quality rating investigating physical activity environment, food environment and T2D.

| Author | Year | Country | Income level | Study design | Sample size | Age | Outcome† | Outcome assessment‡ | Exposure category | Exposure assessment | Level geodata | Quality statement |
| --- | --- | --- | --- | --- | --- | --- | --- | --- | --- | --- | --- | --- |
| Babey et al. (76) | 2008 | US | High | Cross-sectional | 43,000 | > 18y | T2D/T1D prevalence rate | Self-report | Food | GIS | Individual | Weak |
| Ewing et al. (129) | 2014 | US | High | Cross-sectional | 709,234 | > 18y | T2D/T1D prevalence | Blood sample | PA | Secondary | Aggregate | Weak |
|  |  |  |  |  |  |  |  |  |  |  |  |  |
| Herrick et al. (51) | 2015 | US | High | Cross-sectional | 15,522 | 41.0 ± 12.6 | T2D/T1D prevalence | Blood sample | PA, food | Place of residence | Individual | Weak |
| Jiao et al. (77) | 2015 | US | High | Cross-sectional | 2,001 | 54.0 ± 15.0 | T2D/T1D prevalence | Blood sample | Food | GIS | Individual | Weak |
| Marshall et al. (52) | 2014 | US | High | Cross-sectional | 1,044 | 35.5 ± 8.3 | T2D/T1D prevalence | Self-report | PA, food | GIS, environmental audit | Aggregate | Weak |
| Salois et al. (55) | 2012 | US | High | Cross-sectional | NA | NA | T2D/T1D prevalence | Secondary | PA, food | Secondary | Aggregate | Weak |
| Shaffer et al. | 2017 | US | High | Cross-sectional | 444 | 21.3 ± 1.43 | Glycaemic marker: fasting glucose | Blood sample | PA | Self-report | Individual | Weak |

Supplementary Table 2.3: Study results of studies a weak quality rating investigating the association of physical activity environment, food environment or residential noise with T2D.

| Author | Exposure | Study result* | 95% Confidence interval or p-value | At least age and sex adjusted |
| --- | --- | --- | --- | --- |
| Babey et al., 2008 | Food environment: RFEI^¥^   1. RFEI > 5 2. RFEI 3 - 4,9 3. RFEI < 3 | Prevalence:   1. 8.1% 2. 7.8% 3. 6.6% | P < 0.05 (high vs. low RFEI) | No |
| Ewing et al., 2014 | 1. Original sprawl index (density) 2. Refined sprawl index‡ | T-ratio   1. -2.22 2. -2.27 | 1. P < 0.05 2. P < 0.05 | Yes |
| Herrick et al., 2015 | 1. Walkability (per SD change) 2. Supermarket density (per square mile) | OR:   1. 1.19 2. 0.84 | 95%CI:   1. 1.04 – 1.37 2. 0.71 – 0.99 | Yes |
|  |  |  |  |  |
| Jiao et al., 2015 | Distance to closest fast food restaurant | OR: 1.29 | 95%CI: 0.83 – 1.99 | Yes |
| Marshall et al., 2014 | Block group level variables   1. Connectivity variables 2. Intersection density (per square mile) 3. Number of fast food restaurants 4. Number of big box stores 5. Number of grocery stores   City level:   1. Intersection density (per square mile) 2. Average tot number of lanes on major streets 3. Percent of major streets with bike lanes 4. Number of fast food restaurants 5. Number of fitness centres 6. Number of convenience stores | Beta (SE):   1. NR 2. NR 3. NR 4. 0.014 (SE NR) 5. NR 6. -0.0004 7. 0.029 8. -0.07 9. -0.001 10. NR 11. 0.008 | 1. NS 2. NS 3. NS 4. P < 0.10 5. NS 6. P < 0.05 7. P < 0.05 8. P < 0.05 9. P < 0.05 10. NS 11. P < 0.05 | No |
| Salois et al., 2012 | Local food economy:   1. Farmers' market density 2. Direct farm sales per capita (dollars) 3. Percent of farms with direct sales 4. Fast food restaurants density 5. Full-service restaurants density 6. Supermarkets-grocery store density 7. Convenience stores no gas density 8. Convenience stores with gas density 9. Supercentres and club density 10. Recreational and fitness facilities density 11. ERS natural amenity index | Intercept = 9.5, beta:   1. -0.925 2. -0.013 3. -0.007 4. 0.321 5. -0.606 6. -0.002 7. 1.993 8. 0.199 9. 1.69 10. -0.644 11. -0.051 | 1. p < 0.05 2. p < 0.01 3. NS 4. p < 0.01 5. p < 0.01 6. NS 7. p < 0.01 8. NS 9. NS 10. NS 11. NS | No |
| Shaffer et al., 2017 | Walkability:  Males:   1. sidewalks 2. traffic 3. crime during day 4. crime at night   Females   1. sidewalks 2. traffic 3. crime during day 4. crime at night | Correlation:   1. 0.17 2. -0.08 3. -0.09 4. -0.02 5. 0.09 6. 0.28 7. 0.21 8. -0.16 | 1. P > 0.05 2. P > 0.05 3. P > 0.05 4. P > 0.05 5. P > 0.05 6. P < 0.05 7. P < 0.05 8. P > 0.05 | - |
